# Magnetic Milli‐Spinner for Robotic Endovascular Surgery

**DOI:** 10.1002/adma.202508180

**Published:** 2025-09-19

**Authors:** Shuai Wu, Yilong Chang, Sophie Leanza, Jay Sim, Lu Lu, Qi Li, Diego Stone, Ruike Renee Zhao

**Affiliations:** ^1^ Department of Mechanical Engineering Stanford University Stanford CA 94305 USA

**Keywords:** endovascular robot, magnetic robot, Milli‐spinner, robotic surgery

## Abstract

Navigating the complex and high‐flow environment of human vasculature remains a major challenge for conventional endovascular tools and externally actuated tethered systems. While catheter‐based approaches are the clinical standard, their limited steerability and force transmission hinder access to tortuous or distal vessels, especially in the brain. Untethered robotic systems have emerged as a promising alternative for enhanced flexibility and reachability. However, most designs struggle against the high, pulsatile blood flow in human arteries. Here, the study presents a magnetically actuated milli‐spinner robot that overcomes existing limitations in navigating complex and high‐flow vasculature. Capable of swimming at 23 cm·s^−1^ (73 body lengths per second), the milli‐spinner enables rapid, stable navigation through complex vasculature. This performance is driven by its hollow cylindrical structure with integrated helical fins and slits, which together generate a spinning‐induced flow field that enhances propulsion efficiency and allows the robot to maintain stability and control even in dynamic, pulsatile blood flow environments. In addition to its navigation capabilities, the milli‐spinner enables multifunctional treatment, including localized suction and shear for efficient clot removal, targeted drug delivery, and in situ embolization for aneurysm treatment. These features establish the milli‐spinner as a versatile and powerful platform for next‐generation, untethered endovascular interventions.

## Graphical abstract

The magnetic milli‐spinner, with its unique structural design, achieves rapid and stable navigation through complex vasculature at speeds up to 23 cm·s^−1^. It further enables multifunctional treatment, including localized suction and shear for efficient clot removal, targeted drug delivery, and in situ embolization for aneurysm therapy, establishing it as a versatile platform for next‐generation, untethered endovascular interventions.

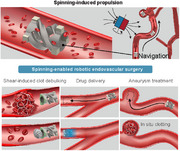

## 1 Introduction

Endovascular devices, including stents and embolization devices, are designed for minimally invasive procedures that rely on X‐ray or other imaging modalities to navigate blood vessels for diagnosing and treating vascular diseases such as atherosclerosis, thrombosis, and aneurysms.^[^ ^1^ ^]^ These devices are typically delivered to the disease site via guidewires and catheters.^[^ ^2^ ^]^ In many cases, however, navigating catheters through highly tortuous regions, such as the cerebral arteries in the brain, remains very challenging. In vessels with sharp turns^[^ ^3^ ^]^ or a large catheter‐to‐vessel diameter ratio,^[^ ^4^ ^]^ there is an increased risk of vessel perforation or dissection. While recent advances in magnetic‐field‐guided or other actuated guidewires and catheters have aimed to improve steerability,^[^ ^5^ ^]^ catheter‐based procedures still face limitations in advancing tethered devices in distal anatomy due to low force transmission to the catheter tip.

To address these challenges, wireless endovascular robots, particularly millimeter‐scale robots, have recently emerged as a promising solution for robotic endovascular surgery.^[^ ^6^ ^]^ These robots offer a practical balance between feasibility, ease of tracking, and controllability compared to their micro‐sized counterparts.^[^ ^7^ ^]^ In these systems, helical‐shaped robots are commonly employed for navigating tubular environments through rotational motion.^[^ ^8^ ^]^ Although studies demonstrate their potential applications, such as drug delivery and blood clot disruption,^[^ ^9^ ^]^ their clinical translation remains significantly constrained. A key limitation is their inability to operate effectively in the high and pulsatile blood flow of human vasculature. Current helical swimmers typically reach speeds of only 0.5–16 cm·s^−1^,^[^ ^8^ ^]^ which is substantially slower than artery blood flow rates. For reference, venous blood flow in the legs averages ≈10–20 cm·s^−1^, while the internal carotid artery (ICA) exhibits flow rates of ≈20–30 cm·s^−1^, with a peak velocity reaching up to ≈60 cm·s^−1^.^[^ ^10^ ^]^ Due to their low swimming speeds, existing helical robots struggle to maintain stable navigation in high‐flow conditions, making it difficult for them to reach the disease site and perform therapeutic functions effectively. Several studies have attempted to overcome high flow by designing robots that generate large friction with vessel walls to enable upstream or downstream motion.^[^ ^6a–c^ ^]^ However, this approach is inefficient in terms of speed, and it risks damaging the vessel walls or triggering spasms that could obstruct blood flow.^[^ ^11^ ^]^

**Figure 1:**
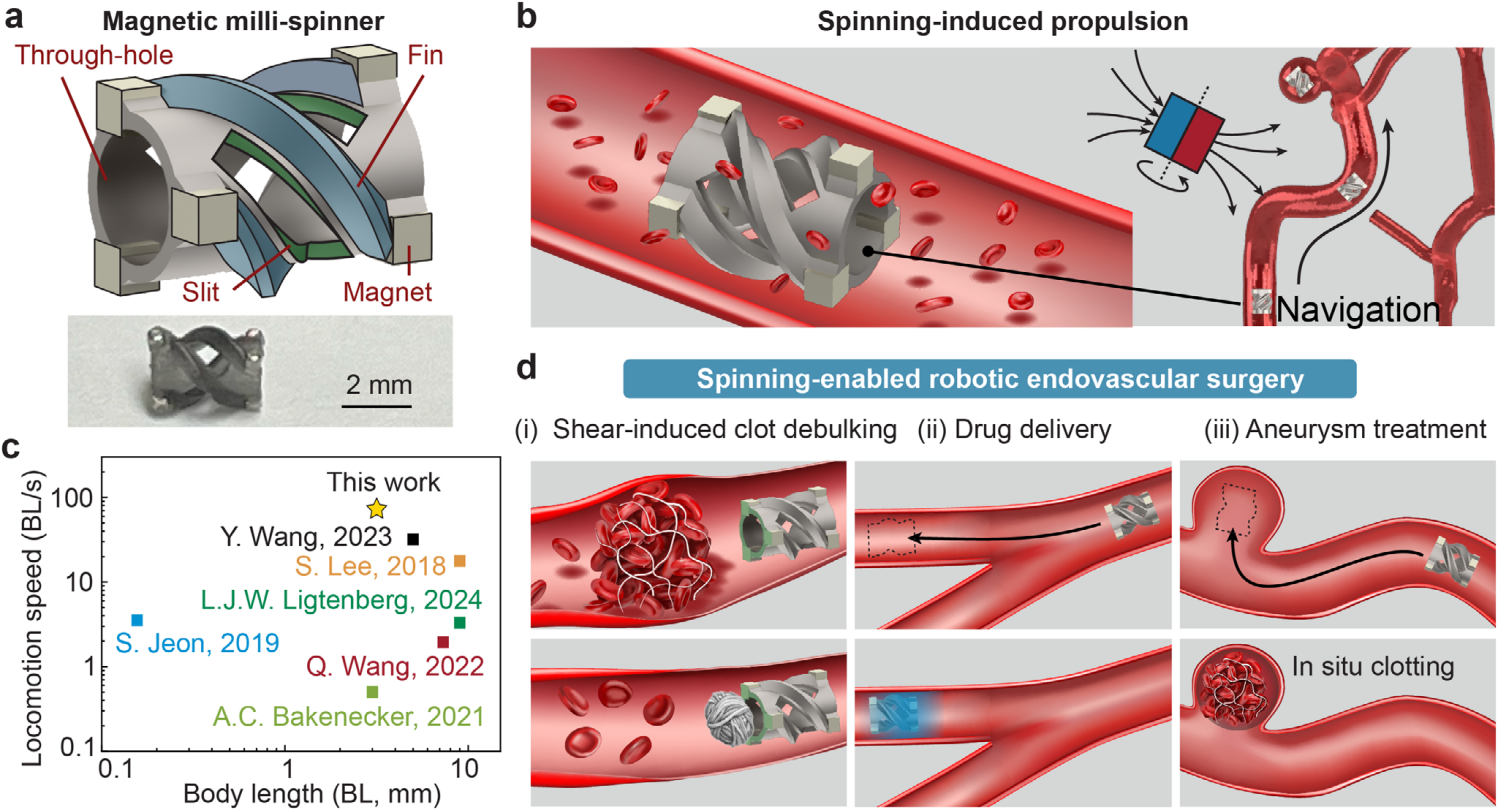
Multifunctional untethered magnetic milli‐spinner robot for minimally invasive endovascular procedures. a) Schematic and photo of the fabricated magnetic milli‐spinner, with key geometric features including a through‐hole, helical fins, and slits. b) Schematic of spinning‐induced propulsion enabling navigation in tortuous blood vessels under a rotating magnetic field. c) Comparison of locomotion performance (normalized speed vs body length) against milli‐robots from literature.^[^ ^6^, ^8^, ^13^ ^]^ d) Demonstration of spinning‐enabled therapeutic functionalities: (i) shear‐induced clot debulking and removal, (ii) drug delivery, and (iii) aneurysm treatment.

Given these limitations, a truly functional wireless endovascular device must integrate two critical capabilities: long‐distance wireless navigation and effective disease treatment in dynamic blood flow conditions. In this work, we introduce a multifunctional magnetic milli‐spinner that achieves both objectives through its novel structural design and its spinning‐induced fluid dynamics (Movie S1, Supporting Information). As illustrated in **Figure** **1**a, the milli‐spinner features a unique hollow cylindrical structure with helical fins and slits (Figure S1, Supporting Information for detailed dimensions), which together maximize propulsion efficiency under a rotating magnetic field for effective navigation (Figure 1b). This unique design enables an unprecedented swimming speed of 23 cm·s^−1^, corresponding to 73 body lengths per second, making it the fastest untethered magnetic robot reported for tubular environments (Figure 1c). Notably, it is the first reported design to achieve swimming speeds comparable to physiological arterial flow rates, which potentially unlocks new capabilities in wireless endovascular robotics.

In addition to its high propulsion speed, the milli‐spinner is engineered with specialized mechanisms that enable multifunctionality and targeted disease treatment. When actuated by external magnetic fields, the milli‐spinner can be precisely navigated to target vascular regions to perform a range of therapeutic functions, including shear‐induced blood clot debulking and removal, drug delivery, and aneurysm treatment (Figure 1d). In particular, during clot treatment, the milli‐spinner generates spinning‐induced localized suction and shear forces that substantially compact the fibrous microstructure of the clot.^[^ ^12^ ^]^ This results in rapid and effective clot debulking, achieving over 90–95% reduction in clot volume within approximately one minute, thereby enabling efficient clot extraction (Figure 1d(i)). Furthermore, the milli‐spinner can precisely deliver therapeutic agents to diseased areas (Figure 1d(ii)) or induce localized clotting for in situ embolization, offering a minimally invasive strategy for treating vascular lesions such as aneurysms (Figure 1d(iii)).

## 2 Results

### 2.1 Magnetic Milli‐Spinner Swimming and Navigation

**Figure 2:**
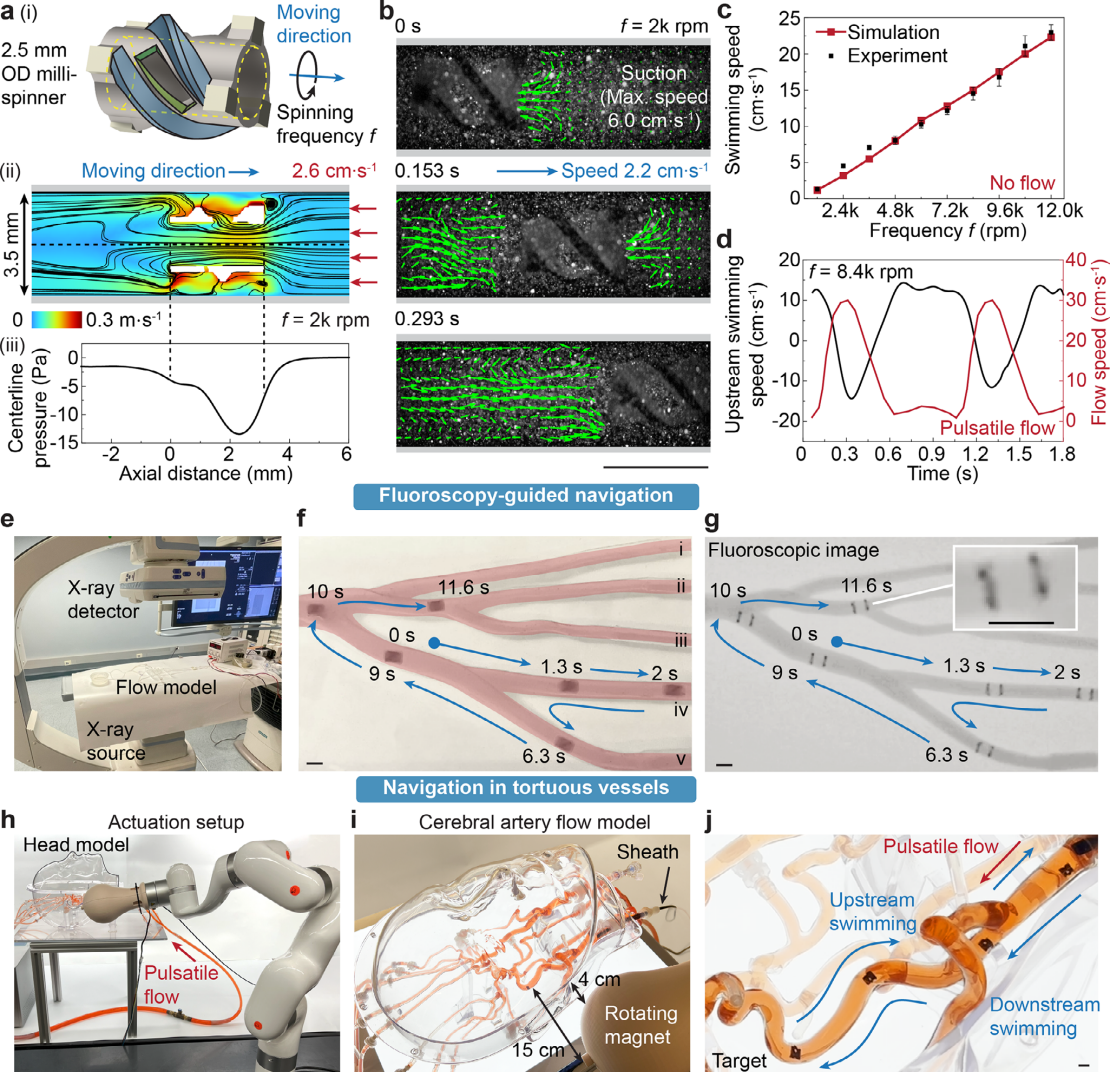
Magnetic milli‐spinner swimming and navigation. a) (i) A 2.5 mm OD milli‐spinner with a through‐hole, fins, and slits. (ii) CFD simulation and streamlines of the milli‐spinner spinning at 2k rpm in a 3.5 mm tube. The hole and slit features induce a flow field in which the flow moves into the hole and either moves out the other end or spins out of the slits. The flow entering the hole results from (iii) the negative pressure generated in the milli‐spinner cavity. b) Micro‐PIV of the milli‐spinner swimming at 2k rpm in a 3.5 mm tube. c) Milli‐spinner swimming speed characterization (mean ± SD) at varied spinning frequencies in a 3.5 mm tube (*n* = 3). d) Milli‐spinner swimming at 8.4k rpm against a pulsatile flow with 30 cm·s^−1^ peak velocity and 60 beats per minute. e) Setup for fluoroscopy‐guided navigation in a pulmonary artery flow model. f) Photo and g) fluoroscopic image showing the real‐time navigation of the milli‐spinner through the pulmonary artery flow model. The zoomed‐in image shows the milli‐spinner, which is visible due to the attached magnets at both of its ends. The magnet is held ≈7.5 cm from the milli‐spinner, generating a rotating magnetic field in the range of 5–10 mT at the milli‐spinner location. h) Control of the milli‐spinner by a robotic arm. i) Experimental setup of a cerebral artery flow model. The magnet is kept around 15 cm from the milli‐spinner, generating a rotating magnetic field in the range of 5–10 mT at the milli‐spinner location and leaving a 4 cm spacing to the head. This distance is sufficient to prevent contact or collision between the milli‐spinner and the magnet during operation, and it can be further increased by utilizing larger magnets or multi‐magnet configurations to maintain the required magnetic field strength at greater distances.^[^ ^15^ ^]^ j) Milli‐spinner navigating in a tortuous vessel with and against the flow. Scale bars: 3.5 mm.

As illustrated in **Figure** **2**a(i), the magnetic milli‐spinner is a cylindrical structure with three key design elements: a through‐hole (dashed yellow line), three helical fins (highlighted in blue), and helical slits (green area). These components are critical for maximizing propulsion to enhance the milli‐spinner's swimming performance and enable additional functions (Movie S2 and Figures S2–S4, Supporting Information for swimming performance comparison of different designs). To investigate the underlying mechanisms of its high swimming speed and therapeutic potential, the spin‐induced fluid dynamics are analyzed both numerically and experimentally for a milli‐spinner with a 2.5 mm outer diameter (OD) operating inside a 3.5 mm diameter tube. As shown in the computational fluid dynamics (CFD) simulation in Figure 2a(ii), the integration of the through‐hole and side slits induces a unique flow field:^[^ ^14^ ^]^ the flow enters through the front opening and exits either through the rear outlet or the side slits. This creates a significant pressure drop within the milli‐spinner cavity (Figure 2a(iii)), which reduces propulsive resistance and increases swimming speed. The combination of through‐hole and slits plays an essential role in enhancing propulsion. Specifically, the through‐hole enables front‐side suction (Figure S3a, Supporting Information), while the slits amplify this suction effect and suppress rear vortex formation to minimize unfavorable energy loss (Figure S2, Supporting Information). Compared to a design with helical fins only, adding a through‐hole increases the swimming speed 1.9‐fold, while incorporating both the through‐hole and slits increases it 3.7‐fold at the same rotation frequency (Figure S2a, Supporting Information). Experimental validation further confirms that the design incorporating both through‐hole and slits achieves the highest swimming speed among all three designs tested (Figure S4, Supporting Information). Additionally, this internal pressure drop also produces an effective suction force, which can be harnessed for functions such as object capture and transport, as well as interaction with blood clots (detailed in the section *Magnetic milli‐spinner for mechanical thrombectomy*). The unique spin‐induced flow field is further validated by micro‐PIV measurements (Figure 2b and see Figure S5, Supporting Information for PIV setup). For optimal visualization and clarity, micro‐PIV is conducted at a reduced spinning frequency of 2k rpm (Movie S2, Supporting Information). At this frequency, CFD predicts a moving speed of 2.6 cm·s^−1^, closely matching the micro‐PIV measured speed of 2.2 cm·s^−1^. The maximum flow speed at the milli‐spinner's front reaches 6.0 cm·s^−1^.

The swimming performance of the 2.5 mm OD milli‐spinner across a broader frequency range (1.2k–12k rpm) is assessed by both CFD simulations and experiments (Figure 2c). Helmholtz coils are employed for generating homogeneous rotating magnetic fields. The results show a linear relationship between spinning frequency and swimming speed, with a good agreement between simulation and measurement. CFD predicts a speed of 58.6 cm·s^−1^ at 30k rpm (Figure S3b, Supporting Information), while a larger 3.5 mm OD milli‐spinner can achieve a simulated speed of 56.4 cm·s^−1^ at just 9.6k rpm spinning frequency (Figure S6, Supporting Information). Experimentally, the 2.5 mm OD milli‐spinner can reach a swimming speed of 14.5 cm·s^−1^ at 8.4k rpm, which is sufficient to swim against a pulsatile flow with a peak flow velocity of 30 cm·s^−1^ (average of 10 cm·s^−1^ or 1 mL·s^−1^), as shown in Figure 2d (more details in Figure S7, Supporting Information). At 12k rpm, it reaches a measured speed of 23 cm·s^−1^. Note that the maximum experimental speed is not reported due to limitations in the spinning frequency range of the employed Helmholtz coil system.

While the milli‐spinner possesses a high swimming speed in a straight tube (Figure 2a–d), it is important to assess the milli‐spinner's performance in clinically relevant vasculatures. Here, utilizing the agile motion control enabled by moving a rotating magnet (see Supporting Information for magnetic actuation setup), the milli‐spinner is navigated through a pulmonary artery flow model under real‐time fluoroscopy guidance (Figure 2e). More details on the experimental setup are provided in Figure S8 (Supporting Information). This flow model offers a relatively simple environment through which the milli‐spinner's steering capabilities can be evaluated, as shown in Movie S3 (Supporting Information). Following the magnetic field's rotational axis, the milli‐spinner can steer and navigate into the target branch at a vessel branching point. As illustrated in Figure 2f, the magnetic milli‐spinner reaches branch (iv) in 2 s. The milli‐spinner can return along the same path by simply swimming in the reversed direction by reversing the spinning direction. Lastly, the milli‐spinner is guided to reach branch (v) at 6.3 s and eventually reaches the branching point of (ii) and (iii) at 11.6 s. Note that the magnets (with a density of 7.6 g·cm^−^^3^) allow for tracking of the milli‐spinner due to their good visibility under X‐ray imaging, even when being obstructed by the human skull (density of 1.6 to 1.9 g·cm^−^^3^).^[^ ^16^ ^]^ As shown in Figure 2g, the milli‐spinner location is evident from the magnets, which are the dark dots at the milli‐spinner's two ends under X‐ray.

The navigation of more complex vasculature, such as the highly tortuous 3D cerebral arteries, requires more precise and sophisticated dynamic control of the milli‐spinner's motion. For these scenarios, a six‐axis robotic arm with an attached rotating magnet can provide multi‐directional control (Figure 2h), enhancing the precision of the milli‐spinner's movement. This robotic system enables milli‐spinner navigation through the intricate and convoluted pathways of the cerebral arteries (Figure 2i) by adjusting the milli‐spinner's orientation in real‐time (Movie S4, Supporting Information). Combining the milli‐spinner's inherent navigation capabilities with the advanced control provided by the robotic arm, the precision required for complex endovascular procedures is achievable. As shown in Figure 2j, the milli‐spinner is first delivered to the cerebral arteries through a sheath and navigates downstream (in the flow direction) to the tortuous vessel in a pulsatile flow of 1 mL·s^−1^ (average of 5 to 10 cm·s^−1^ along the path). Note that when the milli‐spinner is not spinning, it moves very fast, nearly following the flow rate. To keep the milli‐spinner at a controllable speed while moving downstream, it spins at a 5.4k rpm frequency with a propulsion direction that is against the flow direction. While returning, the milli‐spinner can be controlled to swim upstream (against the flow direction) under a higher spinning frequency of 7.2k rpm to effectively overcome the flow. As the milli‐spinner gets close to the sheath, aspiration can be applied to aspirate the milli‐spinner back into the sheath (Movie S4, Supporting Information). This approach could potentially improve the safety of traditional minimally invasive interventions in highly tortuous vasculature. The navigation of the milli‐spinner through the cerebral artery flow model, both forward and backward, is repeated 20 times to demonstrate the reliability of the milli‐spinner using two control methods: a robotic arm‐controlled rotating magnet and a manually controlled rotating magnet. In the robotic system, the arm follows a predefined trajectory, whereas the manual system relies on a trained operator to guide the milli‐spinner. The success criterion is defined as completing the navigation within 1 minute. Both systems achieve a 100% success rate, demonstrating the reliability and repeatability of the milli‐spinner under both robotic and manual control. An additional demonstration showcasing the milli‐spinner navigating through a severely tortuous cerebral artery flow model (with two 360° turns and multiple 180° turns) with manual control is presented in Figure S9 (Supporting Information). Additional simulations and ex vivo experiments, presented in the Supporting Information (Figures S10 and S11, and Movie S5, Supporting Information), demonstrate that the high spinning frequency of the milli‐spinner does not cause vessel damage, including rupture or dissection.

### 2.2 Magnetic Milli‐Spinner for Mechanical Thrombectomy

**Figure 3:**
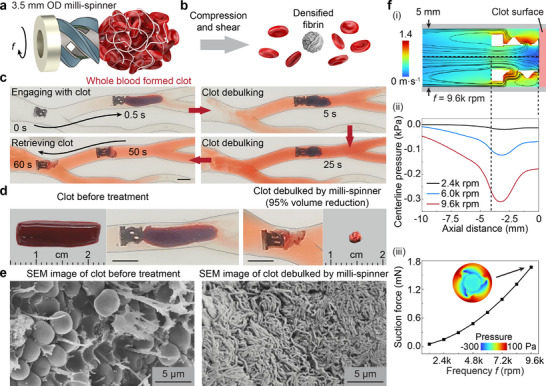
Magnetic milli‐spinner for mechanical thrombectomy. a) Schematics of a magnetic milli‐spinner treating a clot, which is mainly composed of a fibrin network and red blood cells. b) The working mechanism of the magnetic milli‐spinner thrombectomy for clot debulking based on compression and shear to yield a densified fibrin network with significantly reduced size. c) Snapshots of the clot debulking process, which takes only 50 s. d) Comparison of the clot before and after milli‐spinner treatment, illustrating a large clot size reduction to less than 5% of its initial volume. The magnet is kept around 7.5 cm from the milli‐spinner during treatment, generating a rotating magnetic field in the range of 5–10 mT at the milli‐spinner location. e) Scanning electron microscopy images of the clot before (left) and after (right) the milli‐spinner thrombectomy. f) CFD simulation results of (i) the milli‐spinner spinning against an object, (ii) demonstrating a negative pressure in the milli‐spinner cavity, and (iii) a suction force generated on the surface of the object in contact with the milli‐spinner. The suction force is obtained by integrating the pressure on the clot surface. Scale bars: 5 mm unless specified.

Mechanical thrombectomy is a minimally invasive procedure involving mechanical methods, such as using aspiration to vacuum clots and stent retrievers to mechanically cut through and pull clots out from vessels to restore flow.^[^ ^17^ ^]^ As clots can block the flow of blood to vital organs, such as the brain, heart, and lungs, it is important that clot removal can be performed quickly to mitigate permanent tissue damage and cell death. The milli‐spinner shows promise as a device for performing rapid and effective clot removal by directly modifying the microstructure of the clot, which is a fundamentally different mechanism compared to the existing mechanical thrombectomy technologies. A blood clot is primarily comprised of RBCs held within a fibrin network.^[^ ^18^ ^]^ The magnetic milli‐spinner operates by exploiting its magnetic‐field‐driven spinning motion (**Figure** **3**a) to mechanically interact with and remove clots. To demonstrate the concept, a milli‐spinner design with an OD of 3.5 mm is used to operate on a clot in a 5 mm diameter vessel branch in a 1:1 human pulmonary artery flow model. Vessel sizes similar to this are also found in the ICA,^[^ ^19^ ^]^ mid‐superficial femoral vein,^[^ ^20^ ^]^ and renal artery/vein.^[^ ^21^ ^]^ To drive the milli‐spinner thrombectomy, a single ring magnet is utilized as it provides a relatively large magnetic torque (Figure S1c, Supporting Information).

The milli‐spinner thrombectomy mechanism is schematically illustrated in Figure 3b. As the milli‐spinner spins, it generates suction and draws the clot towards its cavity. The localized suction force ensures that the clot is compressed against the milli‐spinner. In the meantime, the spinning motion continuously exerts a shear force on the clot, and the shear can be enhanced by greater suction. The coupled compression and shear applied to the clot leads to the drastic densification and shrinkage of the fibrin network while releasing RBCs (Figure 3b). It should be noted that instead of breaking the clot into small pieces, which causes distal emboli and can lead to severe complications,^[^ ^22^ ^]^ the milli‐spinner modifies the clot structure for significant clot volume reduction (referred to as clot debulking). Mechanical thrombectomy by the magnetic milli‐spinner is demonstrated by debulking a whole blood‐formed clot in a pulmonary artery flow model in Figure 3c. The milli‐spinner navigates to the branch with the clot and successfully engages with the clot within 0.5 s. While spinning at 9.6k rpm, the milli‐spinner debulks the clot to less than 5% of its initial volume within just 50 s. The remaining densified fibrin network is firmly entangled on the milli‐spinner and is removed together with it. Figure 3d shows the size comparison between the red whole blood formed clot before treatment and the densified fibrin residue after treatment. See Movie S6 (Supporting Information) for the experimental in vitro clot debulking. To the best of our knowledge, the demonstrated milli‐spinner clot debulking technology achieves the fastest clot size reduction rate of ≈150 mm^3^·min^−1^ among reported untethered magnetic robots. More importantly, unlike the existing untethered magnetic robots that are based on breaking the clot into small pieces and likely causing distal emboli, the milli‐spinner prevents fragmentation of the clot and distal emboli. The working mechanism is further verified by scanning electron microscopy (SEM), as presented in Figure 3e. The SEM images provide a detailed view of the clot microstructure before and after debulking. Prior to treatment, the fibrin network appears loose and spread out, with most of the clot volume occupied by RBCs. After the milli‐spinner treatment, however, the network is highly compacted and densified, verifying the clot debulking mechanism. This microstructural transformation is critical for reducing the overall size of the clot, facilitating its removal, and minimizing the risk of distal emboli. The milli‐spinner thrombectomy is also demonstrated to effectively treat fibrin clots (Figure S12, Supporting Information), which are hard to address by existing techniques.^[^ ^18^ ^]^

To quantitatively understand the flow field and suction generated by the milli‐spinner that allows for clot debulking, CFD simulations are conducted, with the results shown in Figure 3f. The contour (Figure 3f(i)) visualizes the flow field around the milli‐spinner as it rotates at a frequency of 9.6k rpm against an object (representative of a blood clot) inside a 5 mm diameter tube. The streamlines indicate the presence of a suction toward the milli‐spinner's cavity, and this localized suction behavior is quantitatively measured by the pressure drop along the tube centerline, as shown in Figure 3f(ii). An effective suction force at the milli‐spinner and object interface with respect to different spinning frequencies is calculated by integrating the pressure at the milli‐spinner front, demonstrating that a higher rotational speed leads to more substantial suction forces on the object (Figure 3f(iii)). This suction helps secure the clot against the milli‐spinner's surface and enhances the friction for the densification of the clot, promoting efficient debulking.

### 2.3 Magnetic Milli‐Spinner for Targeted Drug Delivery

**Figure 4:**
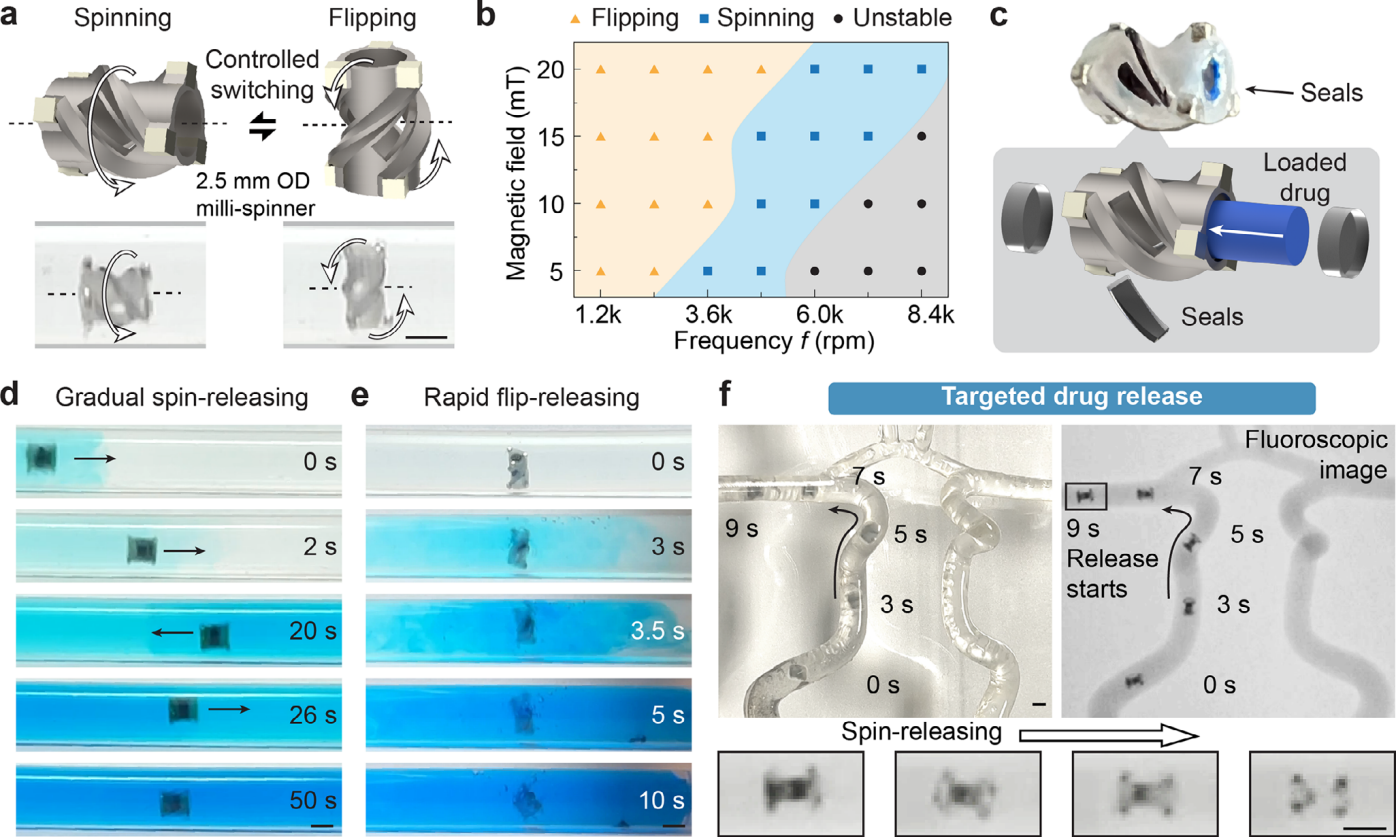
Controlled magnetic milli‐spinner motion for drug release. a) Two distinct motion modes of the milli‐spinner: spinning and flipping, during which the milli‐spinner rotates about the same axis as the rotational magnetic field, indicated by the dashed black line. b) Contour plot illustrating milli‐spinner motion modes under various combinations of magnetic field magnitudes and frequencies (*n* = 3). c) Schematics and fabricated sample of the milli‐spinner loaded with a model drug. d) Snapshots of gradual drug release facilitated by the spinning motion under 20 mT magnetic field and 6k rpm spinning frequency. e) Snapshots of rapid drug release achieved through flipping motion under 20 mT magnetic field and 3.6k rpm spinning frequency. f) Targeted drug release under fluoroscopic guidance. Optical (left) and fluoroscopic (right) images showing the navigation in the cerebral artery flow model. The gradual spin‐releasing starts after the magnetic milli‐spinner reaches the target region. The magnet is kept around 7.5 cm from the milli‐spinner during treatment, generating a rotating magnetic field in the range of 5–10 mT at the milli‐spinner location. Scale bars: 2.5 mm.

In this section, we explore the rotational modes of the milli‐spinner and how switching its rotation axis can be used to control drug release rates for targeted drug delivery. Based on the frequency and the magnitude of the rotating magnetic field, there are two distinct motion modes that the milli‐spinner can achieve, namely spinning and flipping (**Figure** **4**a), with the magnetic field rotating about the dashed black line in the schematic. The milli‐spinner undergoes a spinning motion when it rotates about its longitudinal axis (the axis of the milli‐spinner's cylindrical structure). On the other hand, for the flipping motion, the magnetic field's rotational axis is perpendicular to the milli‐spinner's longitudinal axis (Movie S7, Supporting Information). As shown in the contour plot of Figure 4b, the two motion modes, spinning and flipping, can be switched by adjusting the magnetic field magnitude and frequency. For instance, at 15 mT, the milli‐spinner tends to flip when the rotational magnetic field frequency is relatively low (below ≈3.6k rpm). As the frequency increases to 4.8k rpm, the milli‐spinner transitions into the spinning mode. However, if the frequency becomes too high (above ≈7.2k rpm), the milli‐spinner vibrates in place and cannot stably follow the rotational magnetic field to move. To achieve stable spinning at higher frequencies, stronger magnetic fields are required (e.g., 20 mT for 8.4k rpm spinning).

The different motion modes of the milli‐spinner can be exploited for functionalities such as controlled drug delivery. As shown in Figure 4c, the magnetic milli‐spinner's hollow structure allows for drug storage, with the drug sealed inside by soluble covers. The milli‐spinner can then release the drug at different rates depending on its motion mode under varying magnetic field conditions. Drug release begins with the dissolution of a seal, followed by diffusion of the drug. Simulations (Figure S13, Supporting Information) indicate that the flipping mode leads to faster seal dissolution and drug diffusion, attributed to a higher local fluid velocity (≈0.87 m·s^−1^) near the milli‐spinner ends and a larger effective opening area (3.73 mm^2^). In contrast, the spinning mode generates a lower fluid velocity (≈0.66 m·s^−1^) near the side slits with a smaller opening area (2.53 mm^2^), resulting in slower release. A proof‐of‐concept demonstration for controlled drug release using the milli‐spinner is shown in Figure 4d,e (Movie S7, Supporting Information), in which blue powder is loaded and sealed into the milli‐spinner as a drug model. With a spinning motion, the milli‐spinner gradually releases the drug as it moves back and forth, and the blue color slowly intensifies over time (Figure 4d). Alternatively, when the milli‐spinner flips, the loaded drug is released rapidly in just 5–10 s, as evidenced by the sudden burst of blue color (Figure 4e). The magnetic milli‐spinner's targeted drug release capability is also demonstrated in a 1:1 cerebral artery flow model (Figure 4f; Movie S8, Supporting Information). Dense powders (copper, 40 µm) are loaded and sealed into the milli‐spinner to illustrate the fluoroscopy‐guided targeted drug release process. Initially, the milli‐spinner body is visible due to the dense powder in its cavity, as illustrated in Figure 4f. Following a programmed navigation path, the milli‐spinner reaches the targeted middle cerebral artery (MCA) in 9 s, after which the seals gradually dissolve and release the powder (Movie S8, Supporting Information). The drug release process is also indicated by the progressive fading of loaded powder in the milli‐spinner cavity in the fluoroscopic images, with only the magnets remaining visible once the drug has been fully dispensed (Figure 4f).

### 2.4 Magnetic Milli‐Spinner for Aneurysm Treatment

Aneurysms are weakened, bulging areas of artery walls that pose a significant risk of rupture, leading to severe medical complications, for example, hemorrhagic stroke if occurring in the cerebral artery. Current minimally invasive surgeries for aneurysms rely on interventional procedures that use guidewires and catheters to navigate to the lesion and perform treatment. These procedures, such as aneurysm flow diverter placement or endovascular coiling, aim to achieve selective embolization (in situ clotting) and reduce/prevent blood flow into the weakened area.^[^ ^2^ ^]^ However, current treatment methods can be challenging due to difficulties with catheter/guidewire navigation in complex and tortuous blood vessel anatomy.

**Figure 5:**
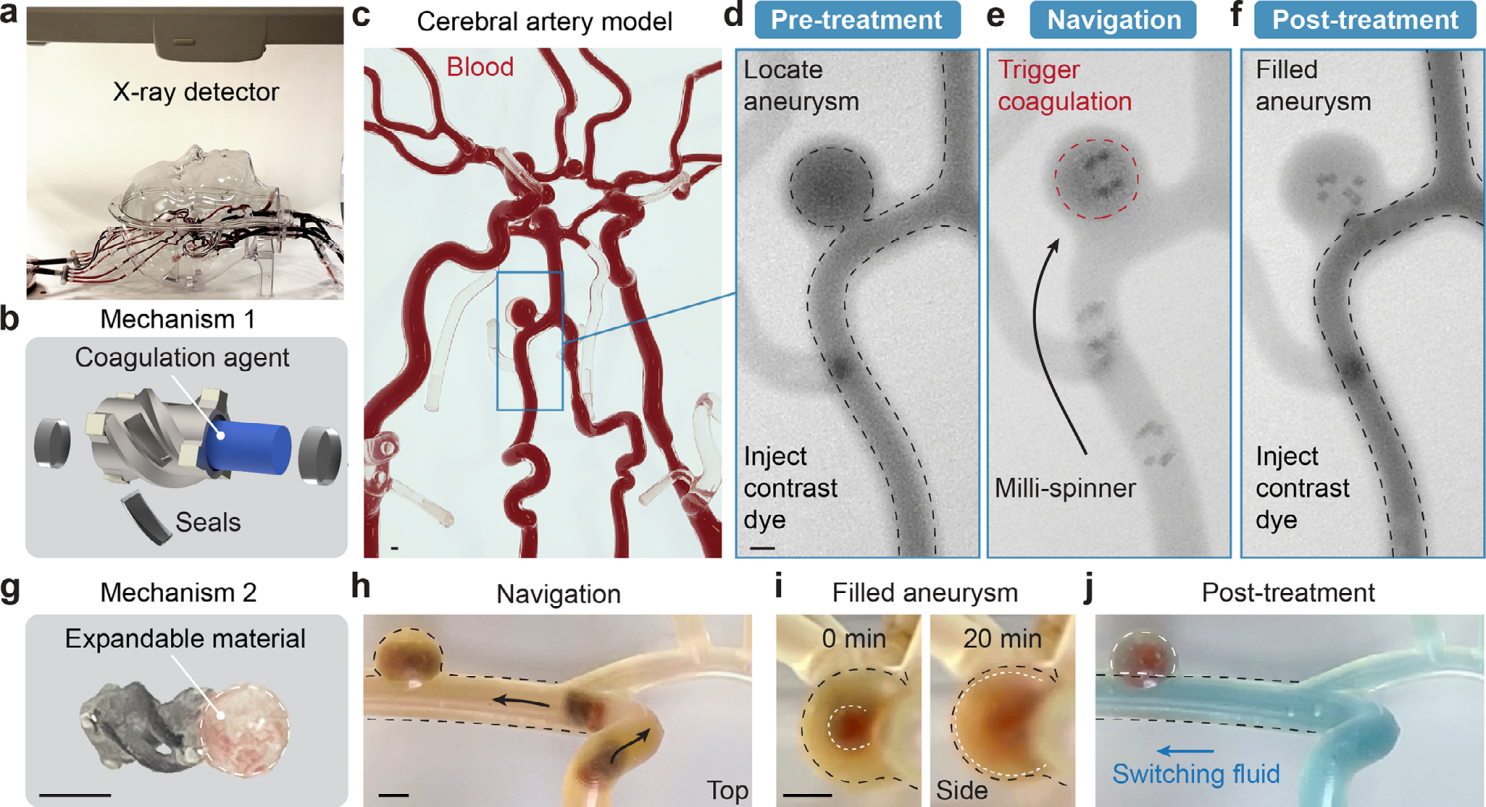
Magnetic milli‐spinner for aneurysm treatment via in situ embolization. a) Experimental setup with fluoroscopic guidance. b) Mechanism 1: milli‐spinner loaded with coagulation agent for in situ embolization. c) Cerebral artery flow model filled with porcine blood with anticoagulant. d) Pre‐treatment angiogram shows the location of the aneurysm. e) Milli‐spinner navigates to the aneurysm and spin‐releases the drug, triggering coagulation in the aneurysm. The magnet is held ≈7.5 cm from the milli‐spinner, generating a rotating magnetic field in the range of 5–10 mT at the milli‐spinner location. f) Post‐treatment angiogram demonstrates the filled aneurysm due to in situ embolization by the milli‐spinner. g) Mechanism 2: milli‐spinner attached with expandable material for in situ embolization. h) Milli‐spinner with attached expandable material navigates to aneurysm location. i) Expandable material attached to the milli‐spinner absorbs water and fills the aneurysm. j) Post‐treatment images showing blocking of blue fluid into the filled aneurysm. Scale bars: 2.5 mm.

In this study, we demonstrate an innovative approach to aneurysm treatment using a magnetic milli‐spinner under fluoroscopic imaging guidance (**Figure** **5**a). The procedure begins with the identification of the aneurysm location via digitally subtracted angiography, a technique that uses X‐ray to obtain the blood vessel map by injecting contrast dye. Once the aneurysm is identified, the milli‐spinner is introduced through a sheath and swims through the tortuous and distal vessels to reach the aneurysm site for embolization. Two different mechanisms for in situ embolization are explored. The first mechanism uses the milli‐spinner to introduce embolic agents (coagulation agents illustrated in Figure 5b) to the targeted lesion. In this demonstration, the cerebral artery flow model is filled with porcine blood with anticoagulant (Figure 5c). After injecting contrast dye, the pre‐treatment angiogram shows a sac structure connected to the regular vessel with a profile denoted by the dashed black line, indicating the aneurysm location, as shown in Figure 5d and Movie S9 (Supporting Information). Under fluoroscopic guidance, the milli‐spinner then navigates into the target aneurysm, where its seals dissolve, releasing the coagulation agent (Figure 5e) to counteract the anticoagulant in the blood, triggering clot formation around the magnetic milli‐spinner within the model aneurysm. Post‐treatment imaging (Figure 5f) shows that the aneurysm is successfully filled, with no contrast dye entering the aneurysm denoted by the dashed black line, confirming effective embolization. Although the milli‐spinner remains within the aneurysm, it would not cause safety concerns after treatment due to its small magnets and weak magnetic force exerted on the clot and surrounding vasculature, even when exposed to strong external magnetic fields. The second mechanism leverages the milli‐spinner's ability to deliver expandable polymeric materials for aneurysm treatment (Figure 5g). As shown in Figure 5h and Movie S10 (Supporting Information), the milli‐spinner carrying an expandable material navigates to the aneurysm. After reaching the target position, the expandable material gradually absorbs liquid, significantly increasing in volume (white dashed line) to fill the aneurysm (black dashed line) as shown in Figure 5i. After the material has fully expanded with the milli‐spinner remaining in the aneurysm, a blue‐colored fluid is flown through (Figure 5j), where it is seen that the blue fluid does not mix with the red material in the aneurysm, indicating reduced flow into the aneurysm. The two presented embolization techniques can serve as powerful and quick methods for targeted aneurysm treatment in tortuous vasculature.

## 3 Conclusion

This study presents a magnetic milli‐spinner, the fastest‐swimming endovascular robot to date, capable of agile navigation in complex vasculature and overcoming high‐flow conditions, enabling a range of robotic endovascular interventions: mechanical thrombectomy, targeted drug delivery, and aneurysm treatment. The milli‐spinner's unique hollow structure with helical fins and slits allows for propulsion upon spinning, resulting in rapid swimming under remote actuation without blocking blood flow. An unprecedented milli‐spinner mechanical thrombectomy technology is introduced, which fundamentally differs from existing state‐of‐the‐art treatments for blood clots. Instead of relying on pulling forces such as those provided by aspiration catheters and stent retrievers, the milli‐spinner applies both compression and shear on the clot. The coupled loadings directly densify the fibrin network and expel RBCs from the clot, enabling effective debulking and complete removal of the clot with minimized risk of distal emboli. In addition, the milli‐spinner can serve as a vessel for targeted drug delivery, accommodating various drug release rates through the control of its motion modes—either spinning or flipping. Lastly, the milli‐spinner shows promise for effective aneurysm treatment in complex and tortuous blood vessel anatomies that are difficult for guidewires and catheters to navigate through.

With the magnetic milli‐spinner's promising navigation capability in complex vasculature and endovascular surgeries, there are a few aspects that should be further studied to improve its abilities. Firstly, CFD simulations can be utilized to optimize the design of the milli‐spinner. As there is an extensive design space, including numerous geometric features of the milli‐spinner as well as different flow conditions and vascular geometries, the milli‐spinner can further be optimized for better performance in different operation environments. Further miniaturization of the milli‐spinner will allow for access to smaller vessels, such as the distal segments of the MCA (M2 and M3 segments). Secondly, in highly tortuous 3D vessels, the robot requires more precise control strategies. Currently, X‐ray imaging informs the position of the milli‐spinner, which dictates where the rotating magnet is moved to for in vitro flow models. However, in reality, real‐time X‐ray imaging will be used for closed‐loop control of the robotic arm and its rotating magnet to keep track of the milli‐spinner's position and guide its orientation for future movements. Accordingly, algorithms can be developed that utilize 2D X‐ray images and map the position of the milli‐spinner to its location within the 3D vasculature, informing on the necessary position of the rotating magnet for the milli‐spinner to reach a desired location or traverse a specific path. This advancement will require highly integrated, multidisciplinary efforts, but it is technically feasible. By leveraging machine learning, a control model can be trained to link the vessel geometry (obtained from CT scans), the milli‐spinner's position and orientation, and robotic arm's movements, ultimately allowing real‐time, image‐guided control of the milli‐spinner targeted intervention. Moreover, deep learning and other advanced control strategies can be effectively integrated to enhance both safety and precision.^[^ ^6^, ^23^ ^]^ For example, preoperative trajectory planning in tortuous anatomy can help identify efficient navigation paths while minimizing risk. Once this is achieved, the milli‐spinner would be able to operate autonomously, which could relieve the burden on interventional radiologists and ensure more reliable medical procedures within tortuous vasculature. We envision that such advancements made to our current technology would lead to more widespread availability of robotic endovascular treatments,^[^ ^24^ ^]^ improving outcomes for patients facing a variety of endovascular conditions.

## 4 Experimental Section

### Magnetic Milli‐Spinner Fabrication

Magnetic milli‐spinners are composed of two components: the main body and attached neodymium iron boron magnets (Figure S1, Supporting Information). The milli‐spinner body was 3D printed using a customized digital light processing printer with a printing resolution of 9 µm. The printer includes a 385 nm UV light projector module (PRO4500, Wintech Digital Systems Technology Corp., USA), a 4× lens (Nikon Corp., Japan), a resin tank with a Teflon AF window (70 µm thickness, VICI Metronics, Inc., USA), and a translation stage (LTS150/M, Thorlabs, USA). The printing resin was prepared with 99.7 wt.% Formlabs Tough 2000 (Formlabs Inc., USA) and 0.3 wt.% iron oxide (300 nm, Alpha Chemicals, USA), which was mixed at 2000 rpm for 30 s (AR‐100, Thinky, USA) to ensure material homogenization. Printing parameters of 2.36 mW·cm^−2^ light intensity, 25 µm layer thickness, and 2.5 s layer curing time are used. Once post‐processing the printed main body, three cube magnets (N50 neodymium, SM Magnetics, USA) were glued to each side for the 2.5 mm OD milli‐spinners in Figures 2, 4, and 5. For the 3.5 mm OD milli‐spinner in Figure 3, one ring magnet (N45 neodymium, SM Magnetics, USA) was attached to the printed structure.

### Magnetic Actuation Setup

Both electromagnetic coils and magnets can provide the magnetic field required to actuate the magnetic milli‐spinner. Different setups of electromagnetic coils (Figure S14, Supporting Information) or motor‐driven magnets (Figure S15, Supporting Information) were adopted for different demonstrations. More details are provided in the Supporting Information *Magnetic actuation setup* section.

### Statistical Analysis

Swimming speed characterization (Figure 2c) was performed on three independently fabricated milli‐spinner specimens. Each specimen was tested five times under identical conditions, and the five measurements were averaged to obtain a representative value for that specimen. The results from the three specimens were then averaged, and the data are reported as mean ± standard deviation (SD). All data processing was carried out using OriginPro 2025 (OriginLab Corp., USA).

Milli‐spinner motion mode contour (Figure 4b) was performed on three independently fabricated specimens. Each specimen was tested five times at each combination of magnetic field magnitude and rotation frequency. The observed motion mode was consistent across all repeated tests.

### Blood Clot Preparation

The porcine whole blood formed clot used in Figure 3c was obtained from Animal Technologies, Inc., USA, and stored in a fridge at 5 °C prior to testing. The milli‐spinner mechanical thrombectomy test was carried out within seven days of the whole blood clot formation, such that there was no noticeable clot degradation.^[^ ^25^ ^]^

The fibrin clot used in Figure S12 (Supporting Information) was fabricated from anticoagulated porcine whole blood with sodium citrate (3.8 wt.%) in the volume ratio of 9:1 from Animal Technologies, Inc. The anticoagulated whole blood was centrifuged at 1100 g for 15 minutes to obtain the citrated plasma. The plasma was coagulated with the addition of calcium chloride (2.45 wt.%) in the volume ratio of 9:1. The mixture was water‐bathed at 37 °C for 1 hour to form the fibrin clots, which were stored in the fridge at 5 °C prior to use.

Porcine whole blood used for clot formation is obtained from a certified commercial supplier (Animal Technologies, Inc., USA) as a slaughterhouse byproduct. No live animal experiment is performed for this study, and the ARRIVE guidelines (Animal Research: Reporting of In Vivo Experiments) do not apply.

### Visualization of Whole Blood Clot and Debulked Clot Microstructures

To visualize the microstructure of the clots before and after treatment using SEM, the clots were first fixed in 0.1 m sodium cacodylate (pH 7.4) with paraformaldehyde (4 vol%) and glutaraldehyde (2 vol%) for one hour, then refrigerated at 5 °C overnight. The next day, the clots were washed with 0.1 m sodium cacodylate buffer (pH 7.4), and post‐fixed in osmium tetroxide (1 vol%) at room temperature for one hour.

After fixation, the clots were dehydrated through a graded ethanol series (50%, 70%, 95%, and twice at 100% ethanol), with each step lasting ten minutes. The clots were then subjected to critical point drying. Once dried, the clots were coated with a thin gold layer for SEM imaging. The images were captured using a Zeiss Sigma SEM (Zeiss Inc., Germany) equipped with a GEMINI electron optical column.

### Milli‐Spinner for Drug Delivery

In Figure 4d–f, food dye or copper particles (40 µm, EnvironMolds, USA), serving as drug mimics, were loaded into the milli‐spinner cavity, followed by sealing of the milli‐spinner with a water‐soluble material (Hypromellose). The filled milli‐spinner was then placed in an oven at 80 °C for one hour to secure the seal.

### In Situ Clotting and Aneurysm Treatment

For the milli‐spinner aneurysm treatment mechanism 1 demonstrated in Figure 5b, anticoagulated porcine whole blood with sodium citrate (3.8 wt.%) in the volume ratio of 9:1 was obtained from Animal Technologies, Inc. Calcium chloride (Aldon Corp., USA), which reversed the effect of the anticoagulant, served as a coagulation agent and was sealed in the milli‐spinner via a water‐soluble material (Hypromellose).

For the milli‐spinner aneurysm treatment mechanism 2 demonstrated in Figure 5g, the expandable material (Polyacrylate) was attached to the milli‐spinner and coated with a water‐soluble layer (Hypromellose) that delays the material's expansion.

## Conflict of Interest

One PCT application has been filed on the reported technology.

## Author Contributions

S.W., Y.C., and S.L. contributed equally to this work. R.R.Z. conceptualized the idea for the study. S.W. and R.R.Z. designed the methodology. S.W., Y.C., S.L., J.S., L.L., Q.L., and D.S. performed the investigation. S.W. performed the visualization. R.R.Z. performed funding acquisition. R.R.Z. performed the project administration. R.R.Z. performed the supervision. S.W., S.L., and R.R.Z. wrote the original draft. S.W., Y.C., S.L., L.L., and R.R.Z. wrote, reviewed, and edited the manuscript.

## Supporting information



Supporting Information

Movie S1

Movie S2

Movie S3

Movie S4

Movie S5

Movie S6

Movie S7

Movie S8

Movie S9

Movie S10

## Data Availability

The data that support the findings of this study are available from the corresponding author upon reasonable request.

## References

[1] S. S. Martin , A. W. Aday , Z. I. Almarzooq , C. A. Anderson , P. Arora , C. L. Avery , C. M. Baker‐Smith , B. B. Gibbs , A. Z. Beaton , A. K. Boehme , Circulation 2024, 149, 347.

[2] P. Schneider , Endovascular Skills: Guidewire and Catheter Skills for Endovascular Surgery, CRC Press, Boca Raton, FL, 2019.

[3] V. Alakbarzade , A. C. Pereira , Pract. Neurol. 2018, 18, 393. 30021800 10.1136/practneurol-2018-001986

[4] R. Prakash , A. Starovoytov , M. Heydari , G. J. Mancini , J. Saw , JACC Cardiovasc. Interv. 2016, 9, 1851. 27609262 10.1016/j.jcin.2016.06.026

[5] a) J. Hwang , J.‐Y. Kim , H. Choi , Intell. Service Robot. 2020, 13, 1;

[6] a) L. Yang , T. Zhang , R. Tan , X. Yang , D. Guo , Y. Feng , H. Ren , Y. Tang , W. Shang , Y. Shen , Adv. Sci. 2022, 9, 2200342; 10.1002/advs.202200342 PMC9165508 35355442

[7] a) N. Li , P. Fei , C. Tous , M. Rezaei Adariani , M.‐L. Hautot , I. Ouedraogo , A. Hadjadj , I. P. Dimov , Q. Zhang , S. Lessard , Sci. Rob. 2024, 9, adh8702; 10.1126/scirobotics.adh8702 38354257

[8] a) A. C. Bakenecker , A. von Gladiss , H. Schwenke , A. Behrends , T. Friedrich , K. Lüdtke‐Buzug , A. Neumann , J. Barkhausen , F. Wegner , T. M. Buzug , Sci. Rep. 2021, 11, 14082; 34234207 10.1038/s41598-021-93323-4 PMC8263782

[9] a) J. Leclerc , H. Zhao , D. Bao , A. T. Becker , IEEE Trans. Robot. 2020, 36, 975;

[10] B. S. Aribisala , Z. Morris , E. Eadie , A. Thomas , A. Gow , M. C. Valdés Hernández , N. A. Royle , M. E. Bastin , J. Starr , I. J. Deary , Hypertension 2014, 63, 1011. 24470459 10.1161/HYPERTENSIONAHA.113.02735 PMC3984108

[11] H. Ishihara , S. Ishihara , J. Niimi , H. Neki , Y. Kakehi , N. Uemiya , S. Kohyama , F. Yamane , H. Kato , Neurol. Med. Chir. 2015, 55, 261. 10.2176/nmc.oa.2014-0268 PMC4533334 25739431

[12] a) Y. Chang , G. Li , J. Sim , G. E. Karniadakis , R. R. Zhao , Ext. Mech. Lett. 2025, 79, 102391;

[13] Y. Wang , H. Chen , J. Law , X. Du , J. Yu , Cyborg Bionic Syst. 2023, 4, 0015. 36939416 10.34133/cbsystems.0015 PMC10019906

[14] Y. Chang , J. G. Vallejo , Y. Sun , R. R. Zhao , Adv. Intell. Syst., 2500609.

[15] Z. Yang , H. Yang , Y. Cao , Y. Cui , L. Zhang , Adv. Intell. Syst. 2023, 5, 2200416.

[16] J. Peterson , P. C. Dechow , Anat. Rec. Part A: Discov. Mol. Cell. Evol. Biol. 2003, 274, 785. 10.1002/ar.a.10096 12923889

[17] J. Gralla , G. Schroth , L. Remonda , K. Nedeltchev , J. Slotboom , C. Brekenfeld , Stroke 2006, 37, 3019. 17053185 10.1161/01.STR.0000248457.55493.85

[18] P. Jolugbo , R. A. Ariëns , Stroke 2021, 52, 1131. 33563020 10.1161/STROKEAHA.120.032810 PMC7610448

[19] J. Krejza , M. Arkuszewski , S. E. Kasner , J. Weigele , A. Ustymowicz , R. W. Hurst , B. L. Cucchiara , S. R. Messe , Stroke 2006, 37, 1103. 16497983 10.1161/01.STR.0000206440.48756.f7

[20] M. G. Annetta , B. Marche , L. Dolcetti , C. Taraschi , A. La Greca , A. Musarò , A. Emoli , G. Scoppettuolo , M. Pittiruti , J. Vasc. Access. 2022, 23, 598. 33749364 10.1177/11297298211003745

[21] L. M. Trunz , R. Balasubramanya , In: StatPearls [Internet], StatPearls Publishing, Treasure Island (FL), 2025, https://www.ncbi.nlm.nih.gov/books/NBK572135.

[22] F. Bala , M. Kappelhof , J. M. Ospel , P. Cimflova , W. Qiu , N. Singh , K. Zhu , B. J. Kim , A. Wadhwa , M. A. Almekhlafi , B. K. Menon , N. Arrarte Terreros , H. Marquering , C. Majoie , M. D. Hill , M. Goyal , ESCAPE‐NEXT Investigators , Stroke 2023, 54, 448. 36689583 10.1161/STROKEAHA.122.040542

[23] a) M. Cai , Q. Wang , Z. Qi , D. Jin , X. Wu , T. Xu , L. Zhang , IEEE Trans. Cybern. 2022, 53, 7699; 10.1109/TCYB.2022.3199213 36070281

[24] Y. Zou , Z. Ren , Y. Xiang , C. Liu , A. Gao , S. Huang , L. Yang , C. Hou , H. Guo , G.‐Z. Yang , Matter 2024, 7, 758.

[25] S. Duffy , M. Farrell , K. McArdle , J. Thornton , D. Vale , E. Rainsford , L. Morris , D. S. Liebeskind , E. MacCarthy , M. Gilvarry , J. Neurointerv. Surg. 2017, 9, 486. 27127231 10.1136/neurintsurg-2016-012308

