# Supplementary material for: Magnetic Milli‐Spinner for Robotic Endovascular Surgery
Source: Adv Mater. 2025 Sep 19;38(4):e08180. doi: 10.1002/adma.202508180 (PMC12810593; doi:10.1002/adma.202508180)
Supplement: Supplementary file 1 — Supporting Information [file ADMA-38-e08180-s010.pdf]

ADVANCED MATERIALS

Supporting Information

for *Adv. Mater.*, DOI 10.1002/adma.202508180

Magnetic Milli-Spinner for Robotic Endovascular Surgery

*Shuai Wu, Yilong Chang, Sophie Leanza, Jay Sim, Lu Lu, Qi Li, Diego Stone and Ruike Renee Zhao**

Supporting Information

Magnetic Milli-spinner for Robotic Endovascular Surgery

*Shuai Wu,[1] Yilong Chang,[1] Sophie Leanza,[1] Jay Sim, Lu Lu, Qi Li, Diego Stone, Ruike Renee Zhao**

Table of content

Supplementary Methods
Supplementary Figures S1-S15
Supplementary Table S1
Supplementary Movies S1-S10

Supplementary Methods

Magnetic milli-spinner swimming mechanism and performance characterization

The magnetic milli-spinner is developed starting from a cylindrical structure with three helical fins (highlighted in blue, **Figure S2a-i**). Structural modifications, including a through-hole (dashed yellow line, **Figure S2a-ii**) and helical slits (green area, **Figure S2a-iii**), are integral to regulating flow around the milli-spinner for better swimming performance (**Movie S2**). From CFD simulation results, **Figure S2a** illustrates the velocity fields around three milli-spinner designs with an OD of ~ 2.5 mm: one with fins only, one with fins and a through-hole, and one with fins, a through-hole, and slits. Here, the velocity contours and streamlines are shown for milli-spinners at 6k rpm spinning frequency in two planes: one along and one perpendicular to a 3.5 mm diameter tube (comparable to the middle cerebral artery size). The milli-spinner behaves similarly to a propeller that can generate linear thrust for propulsion by spinning motion. From the simulation, the milli-spinner with fins only can generate propulsion with an effective moving speed of 2.9 $cm \cdot s^{-1}$ (at 6k rpm spinning frequency) as shown in **Figure S2a-i**. Flow impingement is observed at the milli-spinner front for the design with fins only, corresponding to high resistance against swimming. Additionally, a vortex persists at the back of the milli-spinner, where energy can be wasted in the rotational motion of the vortex instead of being used for forward propulsion. With the through-hole (**Figure S2a-ii**), the milli-spinner's effective moving speed increases to 5.4 $cm \cdot s^{-1}$ at the 6k rpm spinning frequency. From the streamlines in **Figure S2a-ii**, the through-hole enables the fluid entering from the front opening and exiting through the rear outlet of the milli-spinner, accompanied by a reduced vortex region. According to Bernoulli's principle, the high flow speed in the milli-spinner cavity leads to a significant pressure drop (**Figure S2a**), resulting in reduced frontal flow resistance for improved swimming performance, together with a suction force. The adopted milli-spinner design in this work (**Figure S2a-iii**) involves three slits in between the helical fins. It is worth noting that the added slits on the milli-spinner are an important modification that acts as additional outlets allowing flow entered from the front opening to exit, as seen in the velocity field in the plane perpendicular to the tube. Compared to the design with only a through-hole (**Figure S2a-ii**), the milli-spinner with through-hole and slits yields a higher flow speed inside the cavity (**Figure S2a-iii**), resulting in a more significant pressure drop (**Figure S3**). Consequently, the milli-spinner with fins, through-hole, and slits exhibits the highest effective moving speed of 10.8 $cm \cdot s^{-1}$, which is 2 times that of the milli-spinner with the through-hole and fins and 3.7 times that of the milli-spinner with fins only at the same 6k rpm spinning frequency. The velocity contours and streamlines of the same 2.5 mm OD milli-spinners inside a 3.5 mm tube at a relatively slow spinning frequency of 2k rpm are provided in **Figure S2b**, illustrating the same trend of swimming performance. Experimentally, the milli-spinner design with through-hole and slits exhibits the fastest swimming performance among all three designs (**Figure S4**).

Computational fluid dynamics simulations

Computational fluid dynamics (CFD) simulations are carried out in COMSOL Multiphysics 6.1 (COMSOL Inc., USA) to predict the velocity field, swimming speed, and pressure distribution of various magnetic milli-spinners operating in tubular environments. A laminar flow model (Reynolds number smaller than 2200) or k-ε turbulent flow model (Reynolds number larger than 2200) with frozen rotor assumption is implemented to solve the Navier-Stokes equation for Newtonian incompressible flow. For example, in **Figure 2c**, the results of 6k rpm and below are obtained using a laminar flow model, and those above 6k rpm are obtained using a turbulent flow model. In the frozen rotor method, a cylindrical rotating domain which is slightly larger than the milli-spinner is defined, and a rotational velocity along the longitudinal direction of the milli-spinner is specified. Stationary analysis is performed for the fluid flow in the simulations to obtain a quasi-steady flow field. For simulations of the milli-spinner swimming in **Figure 2a**, **Figure S2**, **Figure S3**, **Figure S6**, no-slip conditions are set at the milli-spinner surface and tube wall, zero-pressure is specified at both the inlet and outlet, and a constant moving speed of the tube wall along the longitudinal direction is prescribed. The direction of the prescribed moving speed of the tube wall is opposite to the swimming direction of the milli-spinner, thus simulating the milli-spinner moving forward in the tube. When the milli-spinner is in an equilibrium state, the integration of the milli-spinner surface traction in the longitudinal direction vanishes (less than 10^{-6} N). By iterating the prescribed moving speed of the tube wall until the milli-spinner reaches an equilibrium state, the velocity field, swimming speed, and pressure distribution of the milli-spinner in the tube under a given rotational velocity can be obtained.

For CFD simulations of the milli-spinner interacting with an object (or clot) in **Figure 3f**, to simplify the simulation, the clot blocking the vessel is modeled as a rigid wall, while the vessel is considered as a tube with the milli-spinner positioned nearly in contact with the rigid wall. Either a laminar flow model or k-ε turbulent flow model, depending on the Reynolds number, combined with the frozen rotor method is used to solve the Navier-Stokes equations. The cylindrical region slightly larger than the milli-spinner is defined as the rotating domain, with a specified rotational velocity. A no-slip condition is applied at both the milli-spinner surface and the wall, and a zero-pressure outlet is set as the boundary condition. For all simulations, mesh sensitivity is checked to obtain mesh-independent solutions.

Micro-particle image velocimetry

Micro-particle image velocimetry (Micro-PIV) is utilized as an effective method to experimentally measure microfluidics around the milli-spinner to compare different milli-spinner design performances. As shown in **Figure S5a**, the measurement setup includes a Phantom Miro-4M high-speed camera (Vision Research, Inc., USA) with a 0.6× magnification changer, a Nikon E400 Fluorescence Microscope (Nikon Corp., Japan) with a 2× objective, a 470 nm blue light source SOLIS-470C (Thorlabs, Inc., USA), and a Nikon blue

excitation fluorescence filter cube DM505 B-2A. Here, three milli-spinners with fins only, with fins and a through-hole, or with fins, a through-hole, and slits (2.5 mm OD) freely swim inside a 3.5 mm diameter tube at the same spinning frequency of 2k rpm driven by a 25.4 mm cube magnet 5 cm away from the milli-spinner. The tube is made of fluorinated ethylene-propylene (FEP) for index matching purposes and is surrounded by water in a 50 mm × 10 mm × 10 mm tank (**Figure S5a**). Fluorescent particles (Dragon Green, 1% solid, 15 μm, Bangs Laboratories, Inc., USA) are mixed with deionized water in a volume ratio of 1: 37.5. Raw images (**Figure S5b**) are obtained with 400 μs exposure time and 400 fps frame rate, and processed images using an open source software PIVlab[1] are shown in **Figure S5c**. Agreeing with the CFD simulation in **Figure S2**, for the milli-spinner design with fins only, flow disturbance exists around the milli-spinner as it swims (**Figure S5c-i**), accounting for the slow swimming speed of 0.5 $cm{\cdot}s^{-1}$. In contrast, with the abovementioned modifications, the flow around the milli-spinners is more regulated for both the milli-spinner with fins and the through-hole (**Figure S5c-ii**) and the milli-spinner with fins, the through-hole and slits (**Figure S5c-iii**). A suction is observed at the front of the milli-spinner for both cases, and the introduction of the through-hole and slits enables a higher flow rate of 6.0 $cm{\cdot}s^{-1}$ as compared to that of the milli-spinner with the through-hole, 3.8 $cm{\cdot}s^{-1}$. The higher suction enables the milli-spinner with the through-hole, slits, and fins to have a higher swimming speed (2.2 $cm{\cdot}s^{-1}$) than that with through-hole and fins (1.6 $cm{\cdot}s^{-1}$) or with fins only (0.5 $cm{\cdot}s^{-1}$).

Vessel safety evaluation during milli-spinner operation via simulation and ex vivo testing

To assess whether the high spinning frequency of the milli-spinner could result in vessel injury, such as vessel tearing, dissection or rupture, we have conducted additional simulations evaluating the wall shear stress and pressure generated during operation (**Figure S10**, Supporting Information). Both the simulated wall shear stress and hydrostatic pressure generated by the milli-spinner remain below the reported threshold (40 Pa for wall shear stress[2] and 10-20 kPa for pressure[3]) for vascular injury.

Notably, the milli-spinner avoids direct contact with the vessel wall during operation, which helps prevent damage from high-frequency spinning. As shown in **Figure S10c,** the rotation of the milli-spinner produces a vortex flow field that forms a protective zone between the milli-spinner body and the vessel wall, where the flow pattern around the device leads to outward fluid forces that naturally repel the milli-spinner from the endothelium, keeping it suspended within the lumen. Additional tests in an ex vivo porcine internal carotid artery (4.2 mm inner diameter) further support this: **Figure S11a** shows the milli-spinner travels rapidly through the vessel without causing dissection or rupture. In a separate test, the milli-spinner is held spinning in place at 10k rpm for 2 minutes (**Figure S11b**), and again, no evidence of vessel rupture or dissection is observed.

Magnetic actuation setup

Three-axis Helmholtz coils: custom-built 3D coils, as shown in **Figure S14**, are used to provide a homogeneous rotating magnetic field for the milli-spinner motion characterization without the influence of a magnetic field gradient. Three pairs of Helmholtz coils are arranged orthogonally, and currents can be tuned to control both the magnetic field magnitude and direction. The coils generate 2.7 $mT \cdot A^{-1}$, 3.3 $mT \cdot A^{-1}$, and 3.0 $mT \cdot A^{-1}$ uniform magnetic fields along the *X*, *Y*, and Z axes within an 80 mm × 60 mm × 40 mm working space. The 3D coils are used for **Figure 2c, d**, **Figure 4b, d, e**, and **Figure 5h-j**.

Motor-driven magnet: rotating magnets shown in **Figure S15** are used for the ease of integration with the fluoroscopy imaging system and/or for a larger magnetic field working space (by moving the magnet along a predetermined path). The magnet (25.4 mm or 50.8 mm cube, N52 neodymium, CMS Magnetics, Inc., USA), with a customized holder, attaches to a motor (4690, Pololu Corp., USA) with controllable speed. The 25.4 mm cube magnet setup (**Figure S15a**) is used for **Figure 2f**, **Figure 3c**, **Figure 4f**, and **Figure 5d-e** by moving with a hand, providing a 20-30 mT magnetic field when the magnetic is 5 cm from the magnetic milli-spinner or a 5-10 mT magnetic field at 7.5 cm away (**Figure S15b**). For the head model demonstration in **Figure 2h-j**, the 50.8 mm cube magnet setup (**Figure S15c**) attached to a robotic arm (xArm 6, UFACTORY, China) is adopted for a deeper magnetic field penetration, realizing a 20-30 mT magnetic field at 10 cm away, or 5-10 mT at 15 cm away (**Figure S15d**). The spinning magnet is generally positioned parallel to the milli-spinner within the vascular model and is maintained at a distance of around 7.5 cm or 15 cm, depending on the magnet used. Electromagnetic simulations are carried out in COMSOL Multiphysics 6.1 (COMSOL Inc., USA).

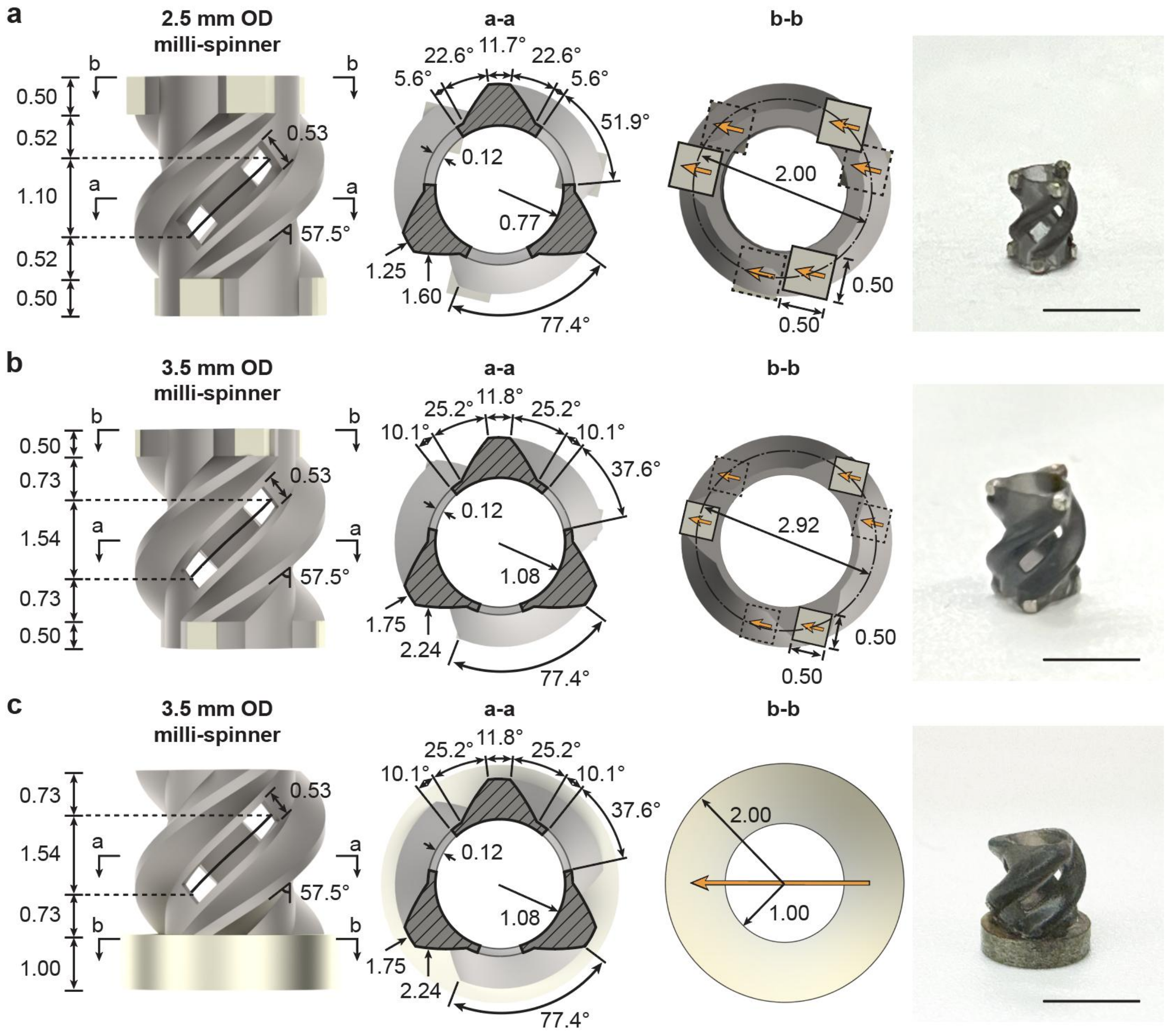

Figure S1. Magnetic milli-spinners of different sizes and designs. (a) Dimensions and image of a 2.5 mm OD magnetic milli-spinner. Three magnets are attached to each side of the magnetic milli-spinner, with their magnetizations denoted by the orange arrows. (b Dimensions and image of a 3.5 mm OD magnetic milli-spinner with three magnets attached to each side. (c) Dimensions and image of a 3.5 mm OD magnetic milli-spinner with a ring magnet. The magnetization is denoted by the orange arrow. Scale bars: 4 mm. All dimensions are given in mm.

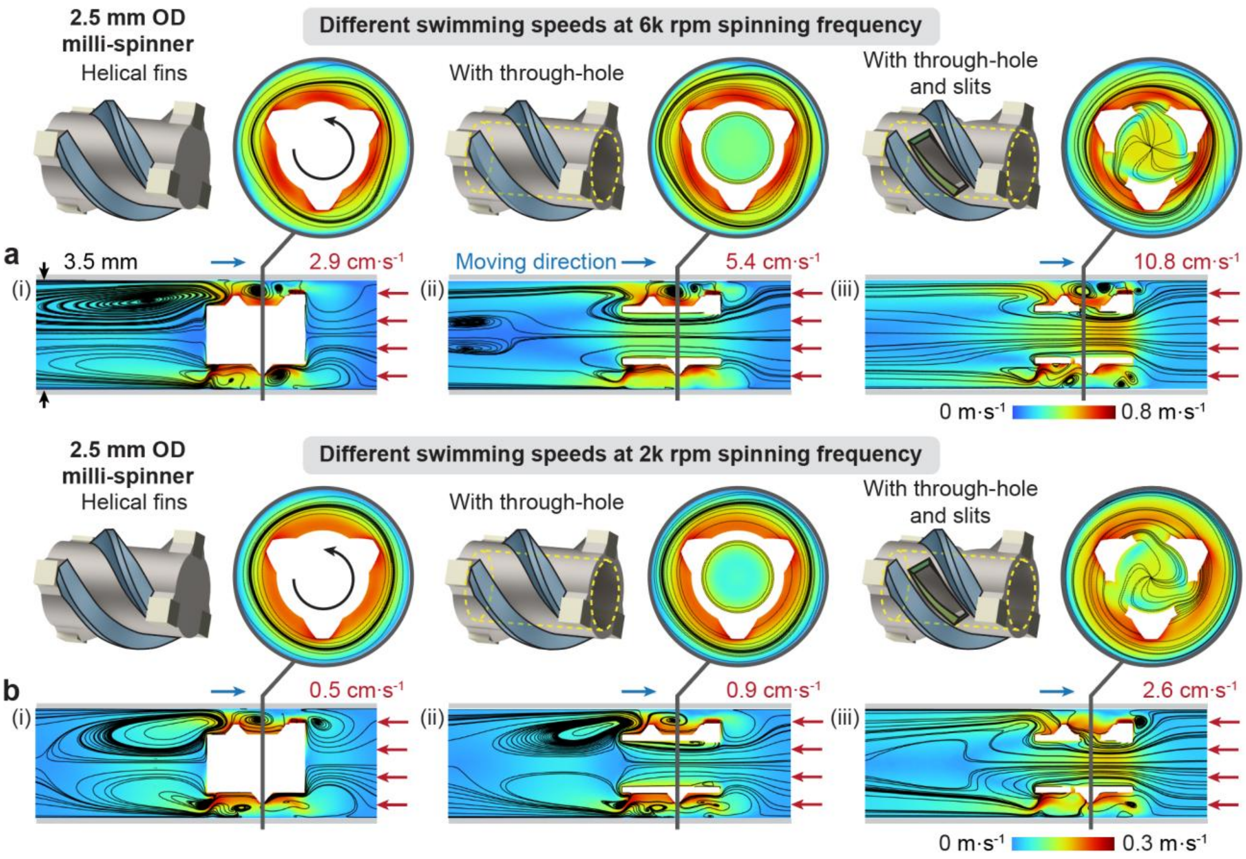

Figure S2. Computational fluid dynamics simulations for performance characterization of various 2.5 mm OD milli-spinner designs. Three 2.5 mm OD milli-spinners with helical fins only, fins and a through-hole, or fins, a through-hole, and slits spinning at (a) 6k rpm and (b) 2k rpm inside a 3.5 mm diameter tube. Structural modifications of a through-hole and slits enable the milli-spinner to stay in equilibrium with a higher moving speed, and thus swim faster, at the same spinning frequency.

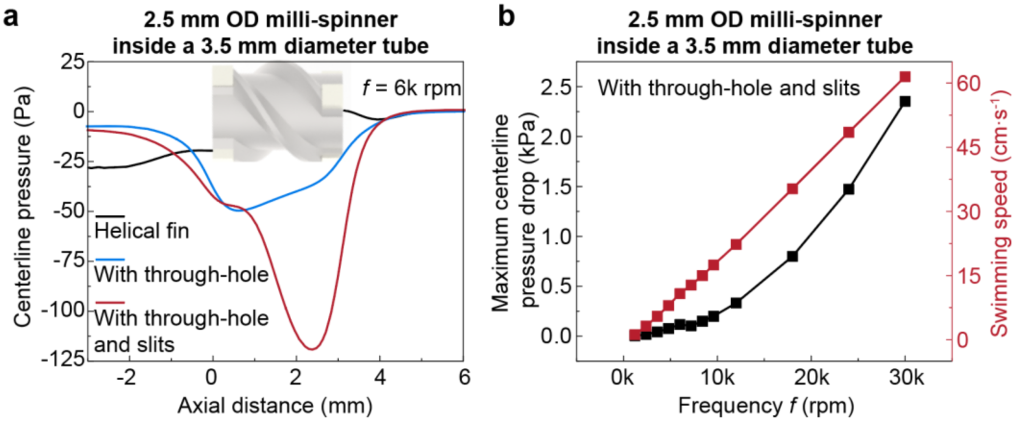

Figure S3. Influences of structural modifications and spinning frequency of the magnetic milli-spinners. (a) Centerline pressure distribution of three 2.5 mm OD milli-spinner designs with helical fins only, fins and a through-hole, or fins, a through-hole, and slits at 6k rpm spinning frequency inside a 3.5 mm diameter tube. (b) Maximum centerline pressure drop and swimming speed of the 2.5 mm OD milli-spinner with fins, a through-hole, and slits at 6k rpm spinning frequency inside a 3.5 mm diameter tube.

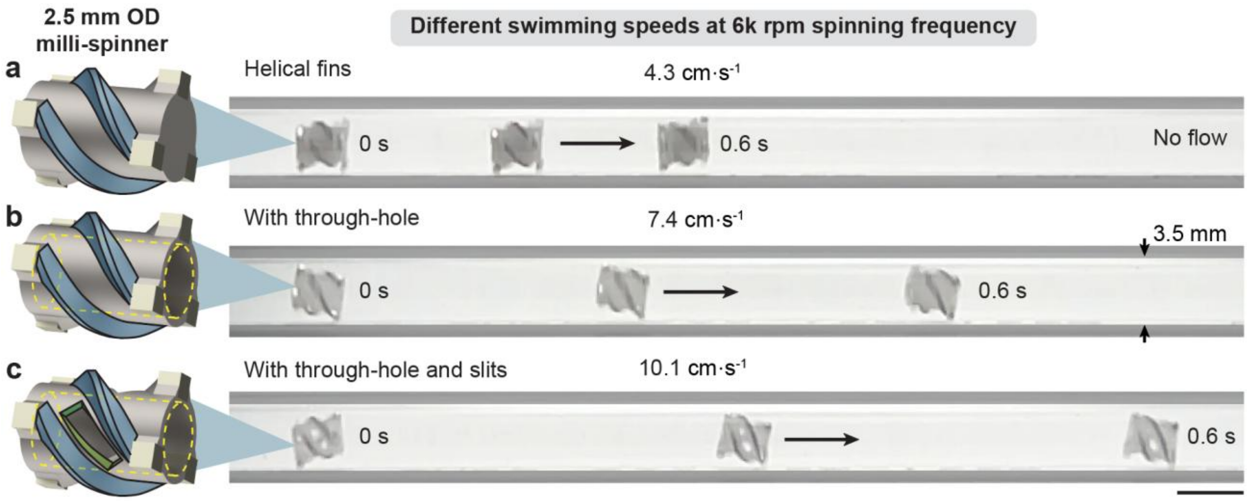

Figure S4. Magnetic milli-spinner structural designs and corresponding swimming performance. Three milli-spinner designs with (a) helical fins only, (b) fins and a through-hole, or (c) fins, a through-hole, and slits show different swimming speeds of 4.3, 7.4, and 10.1 cm·s^{-1} at the same spinning frequency of 6k rpm. Scale bar: 3.5 mm.

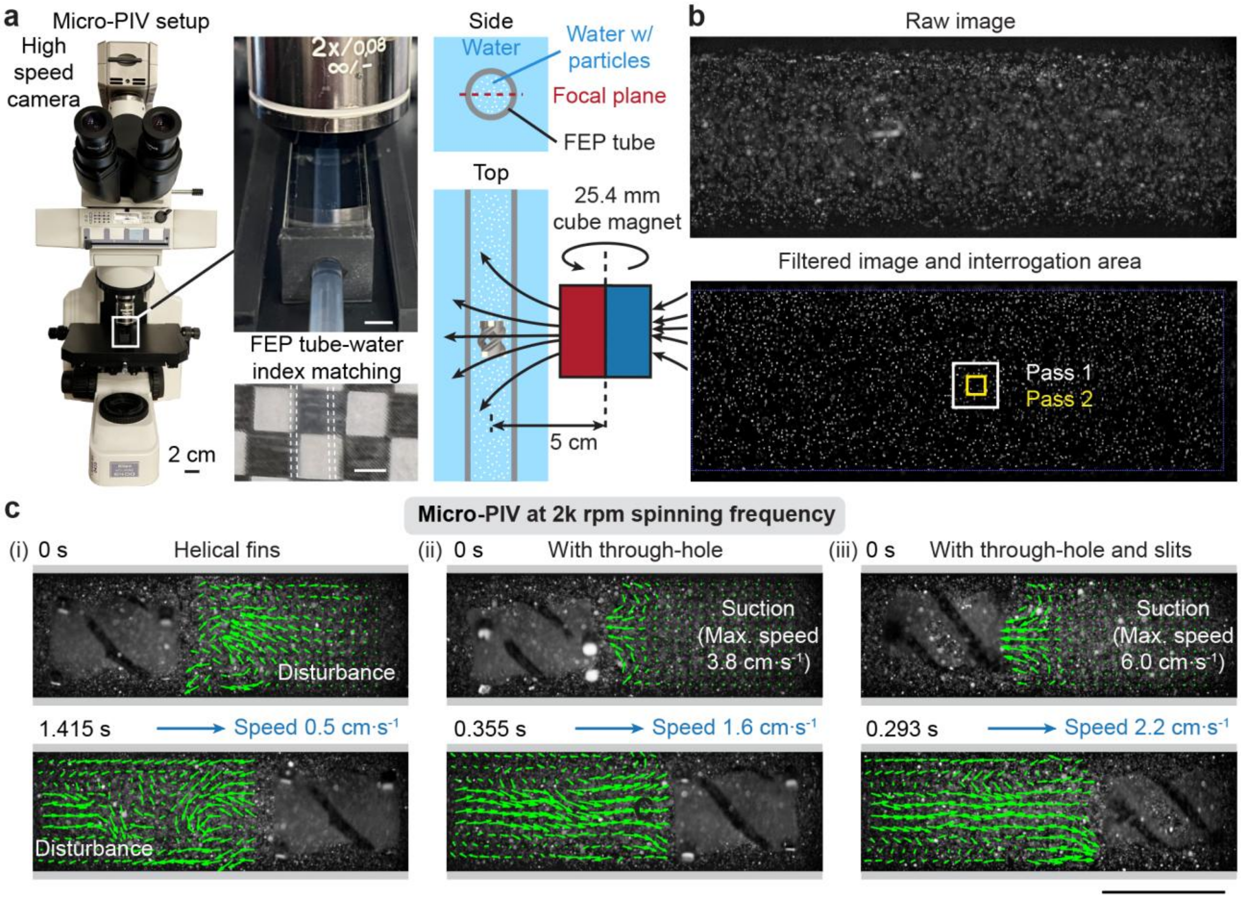

Figure S5. Micro-particle image velocimetry for performance characterization of various 2.5 mm OD milli-spinner designs. (a) Experimental setup including a high-speed camera, a microscope, a 470 nm blue light source, and a 3.5 mm diameter tube in a 50 mm × 10 mm × 10 mm tank. (b) Unprocessed image and filtered image with two passes of interrogation area denoted by the white box and yellow box. (c) Three milli-spinner designs with ~2.5 mm OD spinning at 2k rpm inside a 3.5 mm diameter tube. The milli-spinner with a through-hole and slits achieves a higher moving speed at the same spinning frequency as the others. Scale bar: 3.5 mm.

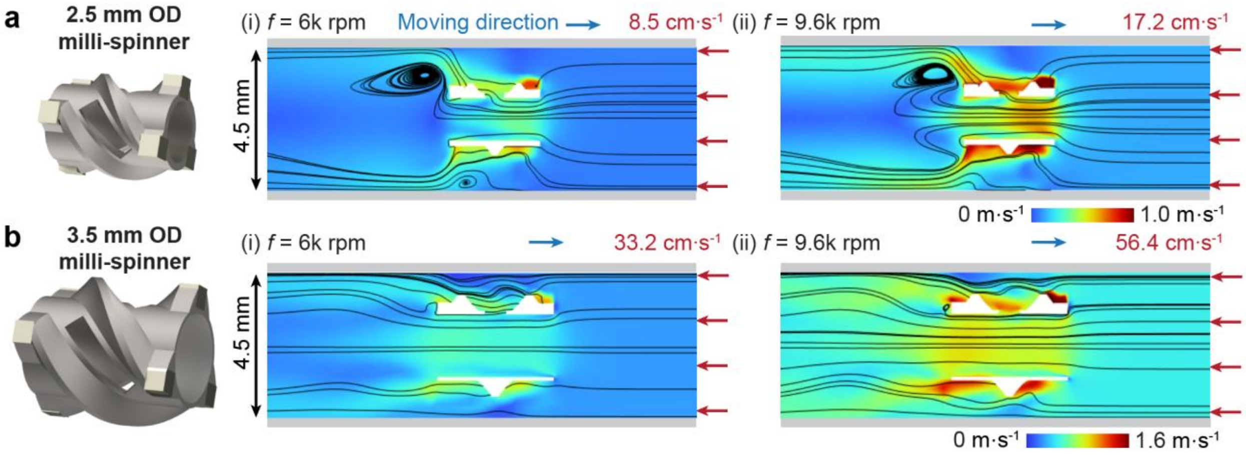

Figure S6. Influences of milli-spinner sizes on the swimming performance. (a) 2.5 mm OD milli-spinner in Figure S1A realizes 8.5 and 17.2 cm·s^{-1} swimming speeds at 6k rpm and 9.6k rpm spinning frequencies. (b) 3.5 mm OD milli-spinner in Figure S1B realizes 33.2 and 56.4 cm·s^{-1} swimming speeds at 6k rpm and 9.6k rpm spinning frequencies.

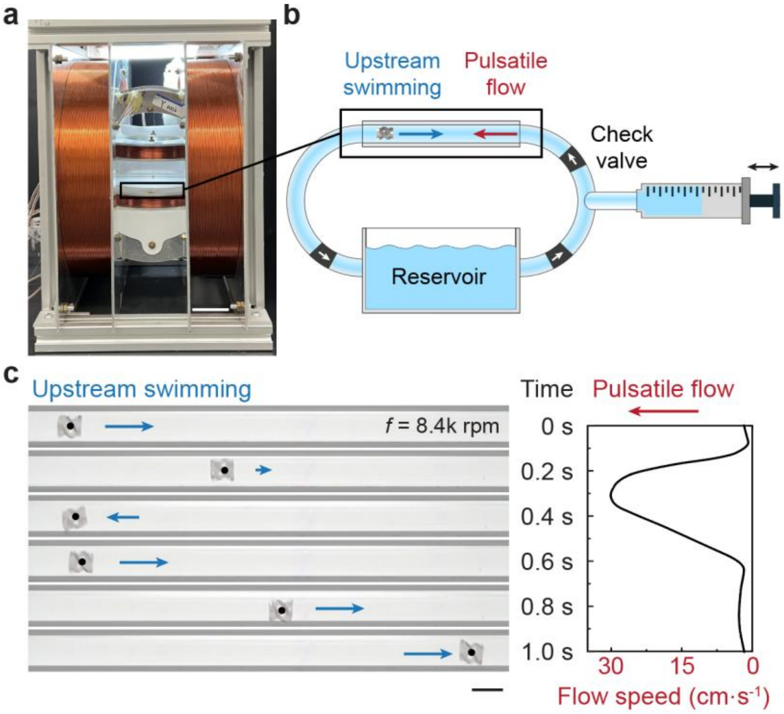

Figure S7. Experimental setup of the milli-spinner swimming against pulsatile flow. (a) Helmholtz coil for a homogeneous rotating magnetic field. Scale bar: 5 cm. (b) Pulsatile flow setup composed of a syringe pump and check valves. (c) Milli-spinner with through-hole and slits spinning at 8.4k rpm for upstream swimming in a pulsatile flow with 60 beats per minute and peak velocity of 30 $cm{\cdot}s^{-1}$. Scale bar: 3.5 mm.

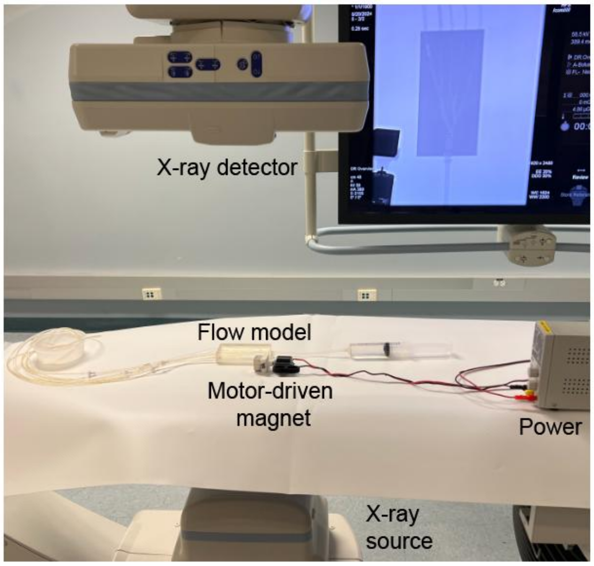

Figure S8. Experimental setup of milli-spinner navigation in pulmonary artery flow model under fluoroscopy guidance.

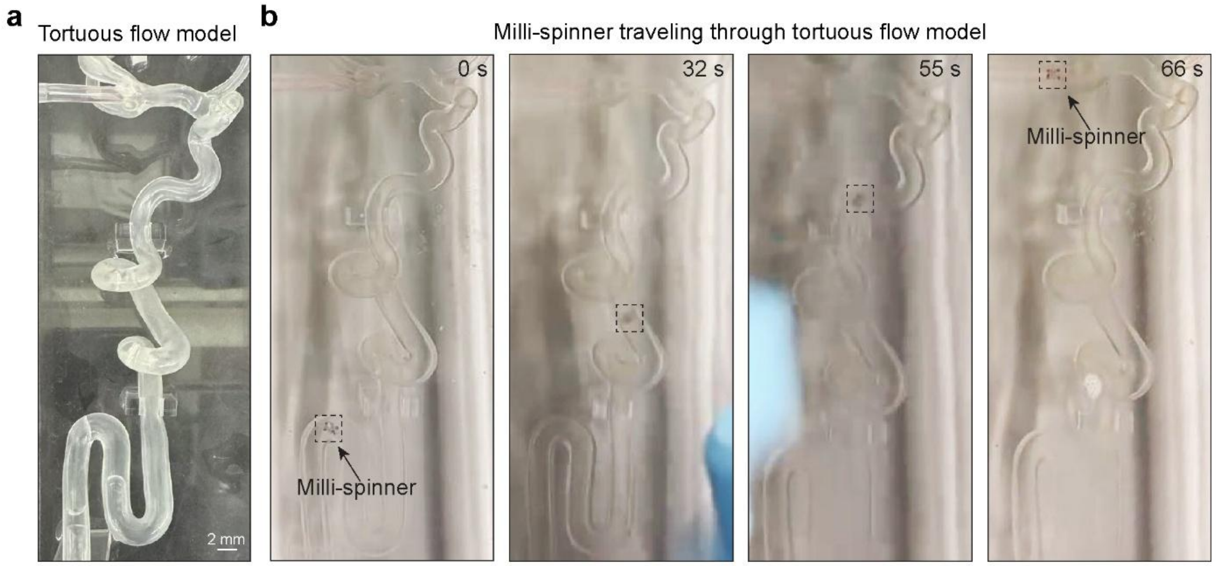

Figure S9. Milli-spinner navigating through a cerebral artery flow model with severe tortuosity, featuring two 360° turns and multiple 180° turns. (a) Picture of the cerebral artery flow model with severe tortuosity. (b) Milli-spinner navigating through the severely tortuous cerebral artery flow model, reaching the first segment of the middle cerebral artery (M1) region from the internal carotid artery (ICA) in 66 s.

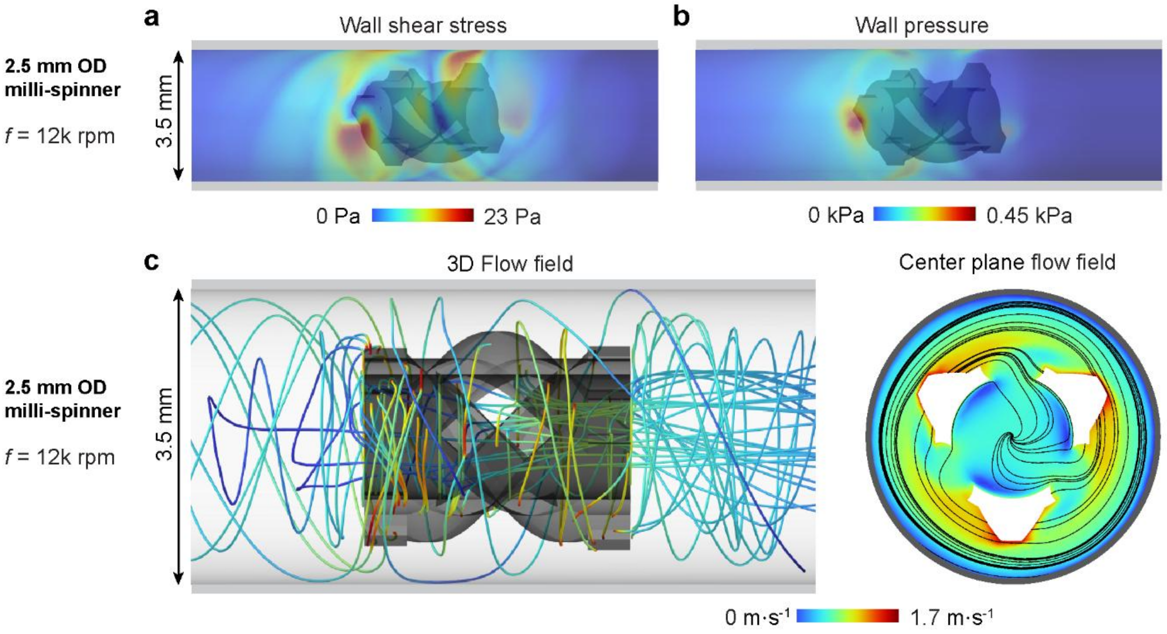

Figure S10. Simulation to evaluate vessel safety during milli-spinner operation. One example of a 2.5 mm OD milli-spinner at a high spinning frequency of 12k rpm is shown. (a) Wall shear stress generated on the tube wall, with peak values up to 23 Pa, which is below the 40Pa threshold that can cause cellular damage or denudation[2]. (b) Wall pressure distribution, remaining below 0.45 kPa, which is below the 10-20 kPa threshold that can cause vessel injury[3]. (c) 3D flow field and the cross-sectional flow field around the milli-spinner, showing surrounding flow that avoids direct contact between the milli-spinner and vessel wall.

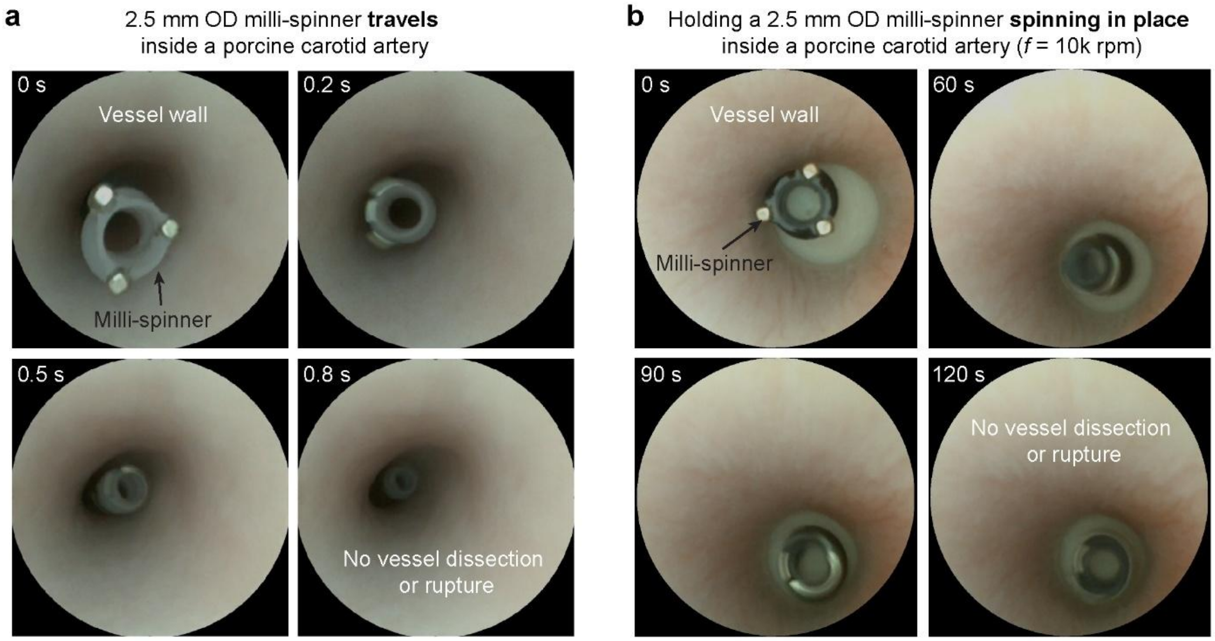

Figure S11. Ex vivo experiments demonstrating no vessel damage during milli-spinner operation. A 2.5 mm OD milli-spinner is tested when it is (a) traveling inside and (b) held spinning at a fixed location for 2 minutes at 10k rpm in a porcine internal carotid artery (~4.2 mm vessel inner diameter).

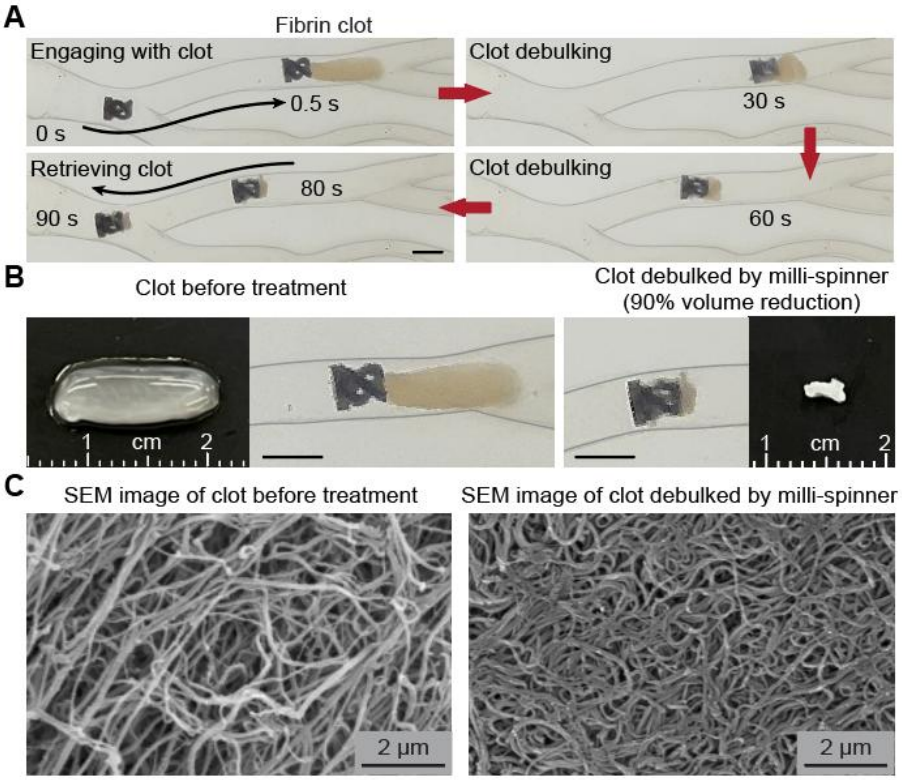

Figure S12. Magnetic milli-spinner for mechanical thrombectomy of fibrin clot. (a) Snapshots of the clot debulking process of fibrin clot, which takes around 80 s. (b) Comparison of the clot before and after milli-spinner treatment, illustrating a large clot size reduction to less than 5% of its initial volume. (c) Scanning electron microscopy images of the clot before (left) and after (right) the milli-spinner thrombectomy. Scale bars: 5 mm unless specified.

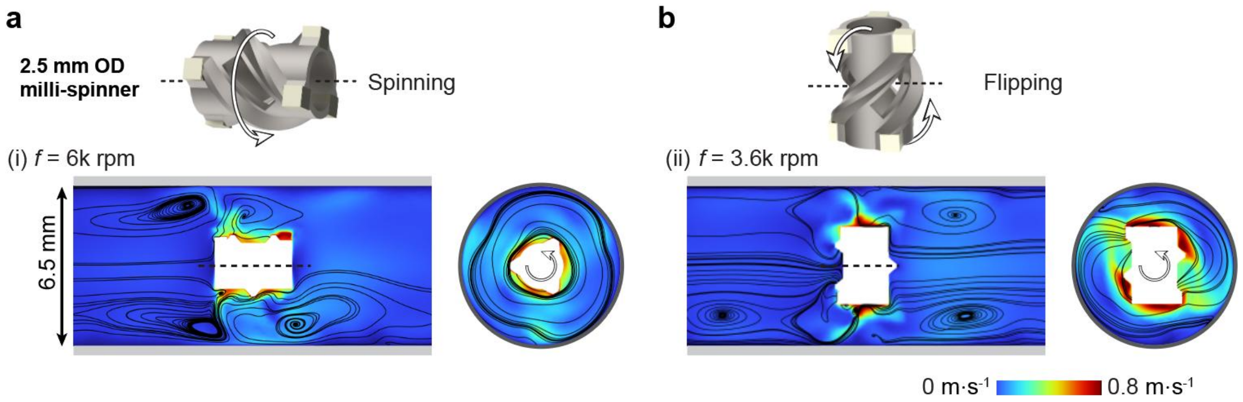

Figure S13. Simulated flow fields of the milli-spinner during (a) spinning at 6k rpm and (b) flipping at 3.6k rpm of the conditions for Figure 4d and 4e. Despite higher frequency, spinning induces lower flow velocity (~0.66 $m \cdot s^{-1}$) near the side slits with a smaller opening area (2.53 mm^2), resulting in slower release. Flipping mode leads to faster seal dissolution and drug diffusion, attributed to a higher local fluid velocity (~0.87 m·s-1) near the milli-spinner ends and a larger effective opening area (3.73 mm^2). Left: longitudinal view; right: cross-section at midplane.

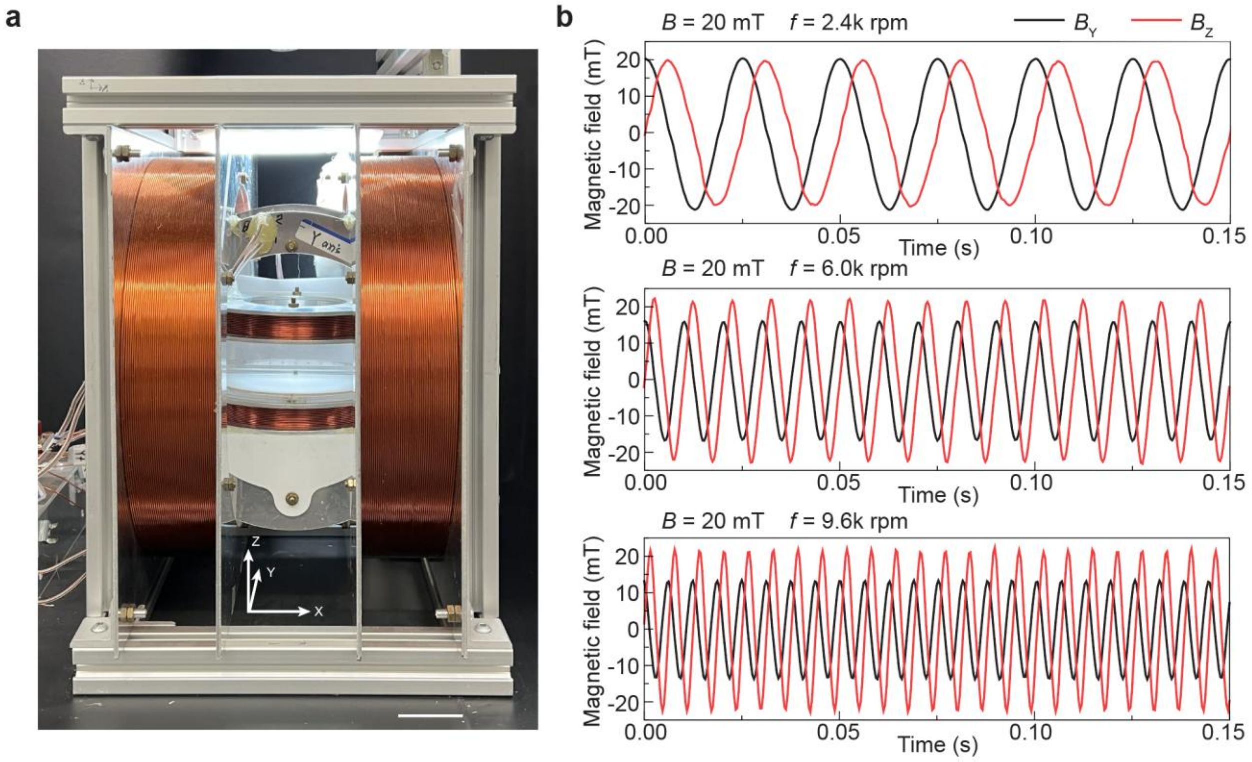

Figure S14. Three-axis Helmholtz coil for a homogeneous rotating magnetic field. (a) Picture of the setup. Scale bar: 5 cm. (b) Magnetic field components at different spinning frequencies of 2.4k rpm, 6.0k rpm, and 9.6k rpm.

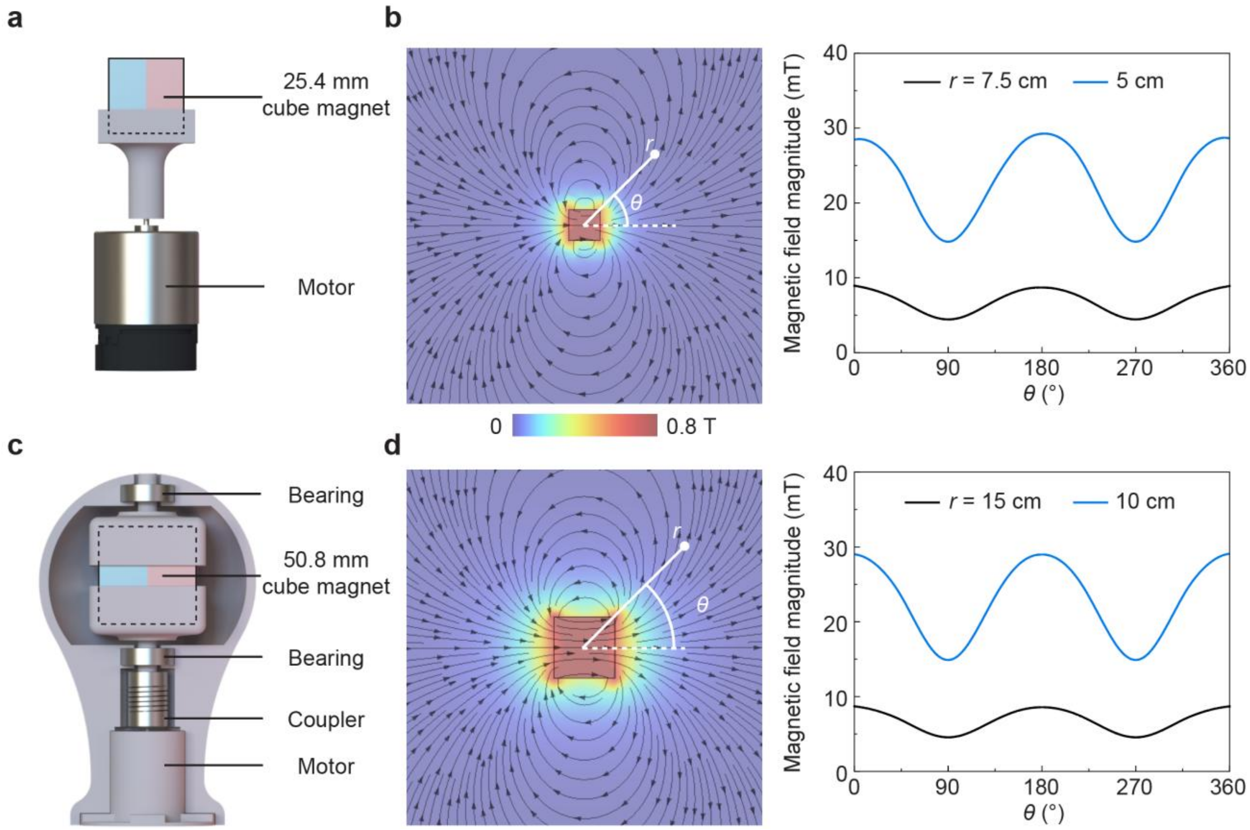

Figure S15. Motor-driven magnets for a rotating magnetic field. (a) Schematic of a 25.4 mm cube magnet setup. (b) Simulated magnetic field magnitude contour of a 25.4 mm cube magnet, which is positioned around 7.5 cm from the milli-spinner to provide a magnetic field of 5-10 mT in corresponding demonstrations. (c) Schematic of a 50.8 mm cube magnet setup. (d) Simulated magnetic field magnitude contour of a 50.8 mm cube magnet, which is positioned around 15 cm from the milli-spinner to provide a magnetic field of 5-10 mT in corresponding demonstrations.

Supplementary Table

Table S1. Comparison of Magnetic Endovascular Milli-robots

Magnetic Milli-robots	Dimension (mm)	Geometry	Motion mechanism	Speed (cm/s)	Control strategy	Potential challenges
Magnetic milli-spinner (This work)	Length: 3.14 Diameter: 2.5	Fin, through-hole, and slit design	Swimming	23	One rotating magnet actuated by a robotic arm	Requirement on control precision, biocompatibility
A. C. Bakenecker, 2021[4]	Length: 3 Diameter: 1.2	Helical Savonius shape	Swimming	0.16	Rotating 3D field from MPI scanner	Vessel safety, biocompatibility
Q. Wang, 2022[5]	Length: 7.3 Diameter: 2.15	Helical shape	Swimming	1.4	Three mobile coils actuated by three robotic arms	Vessel safety, miniaturization
S. Lee, 2021[6]	Length: 9 Diameter: 3	Spiral-shaped driller	Swimming	16	Eight coils (Five-degree-of-freedom system)	Distal emboli from thrombi drilling, in vivo imaging, biocompatibility
S. Jeon, 2019[7]	Length: 0.25 Diameter: 0.08	Helical shape	Screwing Rolling	0.06	Eight coils (Five-degree-of-freedom system)	Vessel safety, navigation in flow, in vivo imaging
Y. Wang, 2023[8]	Length: 5 Width: 0.5	Fish-like streamlined body	Undulating	16	Helmholtz coil	No demonstrated medical functions
L.J.W. Ligtenberg, 2024[9]	Length: 9 Diameter: 3.75	Screw-shaped rigid body	Spinning	3.0	One rotating magnet actuated by a robotic arm	Distal emboli from thrombi drilling, vessel safety

Reference

[1] W. Thielicke, R. Sonntag, *Journal of Open Research Software* **2021**, 9.
[2] a)C. K. Cheng, N. Wang, L. Wang, Y. Huang, *Circulation Research* **2025**, 136, 752; b)D. L. Fry, *Circulation research* **1968**, 22, 165.
[3] a)D. Gonzalez-Rodriguez, L. Guillou, F. Cornat, J. Lafaurie-Janvore, A. Babataheri, E. de Langre, A. I. Barakat, J. Husson, *Biophysical journal* **2016**, 111, 2711; b)D. L. Michell, W. A. Shihata, K. L. Andrews, N. A. Z. Abidin, A.-M. Jefferis, A. K. Sampson, N. G. Lumsden, O. Huet, M.-O. Parat, G. L. Jennings, *Scientific reports* **2021**, 11, 5894.
[4] A. C. Bakenecker, A. von Gladiss, H. Schwenke, A. Behrends, T. Friedrich, K. Lüdtke-Buzug, A. Neumann, J. Barkhausen, F. Wegner, T. M. Buzug, *Scientific reports* **2021**, 11, 14082.
[5] Q. Wang, X. Du, D. Jin, L. Zhang, *ACS nano* **2022**, 16, 604.
[6] S. Lee, S. Lee, S. Kim, C.-H. Yoon, H.-J. Park, J.-y. Kim, H. Choi, *Scientific reports* **2018**, 8, 3691.
[7] S. Jeon, S. Kim, S. Ha, S. Lee, E. Kim, S. Y. Kim, S. H. Park, J. H. Jeon, S. W. Kim, C. Moon, *Science Robotics* **2019**, 4, eaav4317.
[8] Y. Wang, H. Chen, J. Law, X. Du, J. Yu, *Cyborg and Bionic Systems* **2023**, 4, 0015.
[9] L.-J. W. Ligtenberg, N. C. Rabou, C. Goulas, W. C. Duinmeijer, F. R. Halfwerk, J. Arens, R. Lomme, V. Magdanz, A. Klingner, E. A. Klein Rot, *Communications Engineering* **2024**, 3, 68.
